# Real‐World Outcomes of Repeat Ablation Strategies for Atrial Fibrillation: Insights From the Japanese Catheter Ablation Registry

**DOI:** 10.1002/joa3.70200

**Published:** 2025-09-22

**Authors:** Yasuhiro Matsuda, Masaharu Masuda, Koshiro Kanaoka, Toshiaki Mano, Koichi Inoue, Seigo Yamashita, Yu‐Ki Iwasaki, Kohki Nakamura, Koichi Nagashima, Koji Miyamoto, Kazuhiro Satomi, Seiji Takatsuki, Kengo Kusano, Teiichi Yamane, Wataru Shimizu

**Affiliations:** ^1^ Kansai Rosai Hospital Cardiovascular Center Amagasaki Hyogo Japan; ^2^ Department of Medical and Health Information Management National Cerebral and Cardiovascular Center Osaka Japan; ^3^ Cardiovascular Division National Hospital Organization Osaka National Hospital Osaka Japan; ^4^ Department of Internal Medicine, Division of Cardiology The Jikei University School of Medicine Tokyo Japan; ^5^ Department of Cardiovascular Medicine Nippon Medical School Tokyo Japan; ^6^ Division of Cardiology Gunma Prefectural Cardiovascular Center Maebashi Japan; ^7^ Division of Cardiology, Department of Medicine Nihon University School of Medicine Tokyo Japan; ^8^ Department of Cardiovascular Medicine National Cerebral and Cardiovascular Center Osaka Japan; ^9^ Department of Cardiology Tokyo Medical University Tokyo Japan; ^10^ Department of Cardiology Keio University School of Medicine Tokyo Japan

**Keywords:** additional ablation, atrial fibrillation, catheter ablation, repeat ablation, re‐pulmonary vein isolation

## Abstract

**Background:**

Repeat ablation is often required in patients with atrial fibrillation (AF) due to recurrent arrhythmias. Although pulmonary vein isolation (PVI) is the only recommended ablation technique for repeat ablation, various additional strategies are commonly used in clinical practice. The purpose of this study was to evaluate the implementation, efficacy, and safety of repeat ablation strategies in Japan.

**Methods:**

This study was conducted by using the Japanese Catheter Ablation Registry (J‐AB registry). A total of 26 684 patients who underwent a second ablation procedure for AF between August 2017 and December 2020 were included and analyzed for patient characteristics, procedural characteristics, and complications. Additionally, the AF recurrence rate over a 12‐month follow‐up period was also investigated in 1508 s ablation procedures.

**Results:**

In the second ablation procedure, repeat‐pulmonary vein isolation (re‐PVI) was performed for 20 938 (78%) patients and 14 552 (55%) patients underwent left atrial additional ablation. Both of re‐PVI and left atrial additional ablation were performed for 10 086 (38%) patients. As the number of left atrial additional ablations in the second ablation procedure increased, the overall complication rate also significantly increased (paroxysmal AF, *p* < 0.001; persistent AF, *p* < 0.001). The rate of freedom from AF recurrence during the follow‐up period was 87.6% for paroxysmal AF and 80.6% for persistent AF.

**Conclusions:**

In the second ablation procedure performed in Japan, re‐PVI was required in 78% of patients, and both of re‐PVI and left atrial additional ablation were performed for 38% of patients. As the number of left atrial additional ablations increased, the overall complication rate also increased.

**Trial Registration:**

The J‐AB registry is registered in the UMIN Clinical Trial Registry (UMIN 000028288) and ClinicalTrials.gov (NCT03729232)

## Graphical abstract

Patients who underwent second or later ablation procedures for AF were analyzed. In total, 41% of patients underwent re‐PVI only; conversely, both of re‐PVI and left atrial additional ablation were performed for 38% of patients. As the number of additional ablations increased, the overall complication rate also increased.

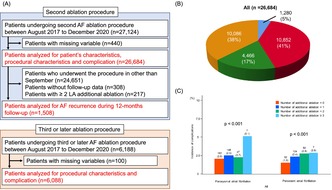

## 1 Introduction

Catheter ablation is an effective treatment for atrial fibrillation (AF) [1, 2]. In the initial ablation procedure for AF, pulmonary vein isolation (PVI) is strongly recommended irrespective of AF duration [1, 2]. However, evidence for the efficacy of additional ablation methods other than PVI is scarce [3, 4, 5].

Because catheter ablation has a substantial risk of AF recurrence after the initial ablation procedure [1, 2], some patients require two or more ablation procedures, or in other words, repeat ablation procedures. Although repeat‐pulmonary vein isolation (re‐PVI) is recommended in cases with pulmonary vein reconnection in repeat ablation [1], the efficacy of additional ablation other than PVI has not been clarified, and current guidelines offer no recommended strategy for additional ablation. In addition, new findings, technological developments, and terminology for additional ablation appear frequently, raising the possibility that additional ablation strategies may differ across operators and institutions.

The Japanese Catheter Ablation Registry (J‐AB registry) is a multicenter, prospective, national cohort registry in Japan [6]. Here, we investigated real‐world data from this registry to evaluate the usage, efficacy, and safety of re‐PVI and additional ablation in repeat ablation procedures in Japan.

## 2 Methods

### 2.1 Patients

The J‐AB registry is a multicenter, prospective, observational cohort registry established to investigate data on real‐world catheter ablation in Japan. The registry is operated by the Japanese Heart Rhythm Society in collaboration with the National Cerebral and Cardiovascular Center using a Research Electronic Data Capture (RED Cap) system [5]. The Japanese Heart Rhythm Society recommends that all catheter ablation procedures performed in Japan be registered and surveyed in the J‐AB registry. Enrollment was started in August 2017, and this study used currently fixed data registered from August 2017 to December 2020.

The design of this study was a subanalysis of the J‐AB registry, which is a multicenter, prospective, observational cohort registry. Inclusion criteria of this study were patients who underwent a second or later catheter ablation procedure, and exclusion criteria were patients who did not agree to participate in the J‐AB registry.

Data on patient characteristics, ablation procedure characteristics, target arrhythmia, acute outcomes, and acute complications were collected from all registered cases. Additionally, detailed ablation data, such as medical materials and rhythm outcomes after the procedure, were also collected for cases performed only in each September of the study period (September cohort).

**FIGURE 1:**
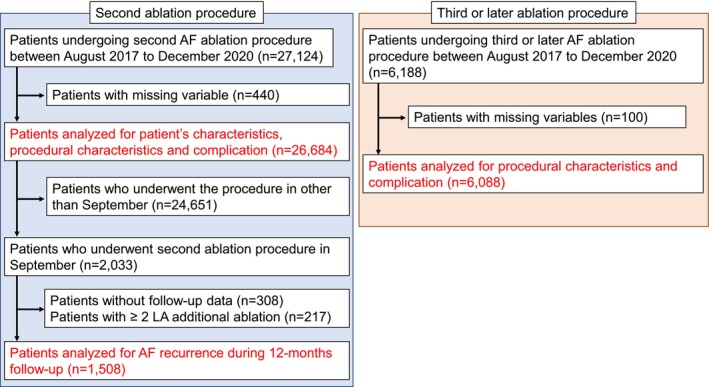
Patient flowchart. From the J‐AB registry, patients with missing variables were excluded, and 26 684 patients who underwent second ablation procedure for AF and 6088 who underwent third or later ablation procedure for AF were analyzed with regard to patient characteristics, procedural characteristics, and complications. After excluding patients without follow‐up data and patients who underwent more than one additional ablation, 1508 patients who underwent second AF ablation procedures during any September in the study period were analyzed for AF recurrence. AF, atrial fibrillation; LA, left atrial.

The patient flowchart for this study is shown in Figure 1. In total, 27 124 patients who underwent the second ablation procedure for AF and 6188 patients who underwent the third or later ablation procedure for AF were included. After excluding patients with missing variables, 26 684 patients who underwent the second ablation procedure for AF and 6088 patients who underwent the third or later ablation procedure for AF were analyzed.

Based on previous guidelines, paroxysmal AF (pAF) was defined as AF that stops spontaneously or without cardioversion within 7 days of onset [1, 2, 7].

This study complied with the Declaration of Helsinki. Written informed consent for ablation and written informed consent or an opt‐out arrangement for participation in the study was obtained from all patients. The protocol was approved by the Institutional Review Board of the National Cerebral and Cardiovascular Center (M28‐114‐7; approved December 2016), as well as by the institutional review boards of all participating hospitals [6]. The J‐AB registry is registered in the UMIN Clinical Trial Registry (UMIN 000028288) and ClinicalTrials.gov (NCT03729232) [6].

### 2.2 Repeat Ablation Procedure

Data on patient characteristics were collected before repeat ablation via the RED Cap system. Re‐PVI was defined as de novo PVI or ablation to the reconnection of the pulmonary veins in repeat ablation. Left atrial additional ablation was defined as ablation other than PVI; however, the AF trigger induction protocol differs from institution to institution, and in Japan, cavo‐tricuspid isthmus linear ablation is already performed as an empirical substrate modification in many cases of the initial ablation procedure [8]; therefore, this definition excluded cavo‐tricuspid isthmus linear ablation and trigger ablation, such as focal ablation for non‐pulmonary vein foci and superior vena cava isolation.

Left atrial additional ablation was divided into six groups: (1) left atrial linear ablation; (2) complex fractionated atrial electrogram (CFAE) ablation (including defragmentation); (3) ganglionated plexi (GP) ablation; (4) rotor ablation (including driver ablation); (5) low voltage area (LVA) ablation; and (6) other left atrial additional ablation [1, 2, 4, 5, 7, 9, 10, 11]. In subgroup analysis, left atrial linear ablation was divided into six groups: (1) left atrial roof line; (2) anterior mitral isthmus line; (3) posterior mitral isthmus line; (4) left atrial posterior wall isolation, that is, left atrial roof line and left atrial floor line; (5) left atrial roof line and anterior mitral isthmus line or posterior mitral isthmus line (roof + MI); and (6) other linear ablation.

### 2.3 Outcomes of Second Ablation Procedure

As a safety outcome, complications in the periprocedural period of the second ablation procedure and third or later ablation procedures were assessed and defined as a composite of hemorrhagic complications, including access site hematoma, cardiac tamponade/effusion, thromboembolism, esophageal complications, gastric hypomotility, atrioesophageal fistula, pericarditis, sick sinus syndrome, atrioventricular block, phrenic nerve paralysis, cardiac artery injury/stenosis, cardiac valve injury, pneumothorax, infection, heart failure, and other complications requiring treatment [6]. In particular, cardiac tamponade/effusion, thromboembolism, and esophageal complications were individually assessed. Esophageal complications were defined as esophageal erosion or ulcer.

Complication rates in patients undergoing a second ablation procedure were analyzed according to ablation strategy. Patients who underwent a second ablation procedure were divided into four groups: (1) patients who underwent neither re‐PVI nor left atrial additional ablation; (2) patients who underwent re‐PVI only; (3) patients who underwent left atrial additional ablation only; (4) patients who underwent both of re‐PVI and left atrial additional ablation. As a subgroup analysis, complication rates in patients undergoing each type of ablation strategy and left atrial linear ablation were also calculated. Complication rates stratified by the number of left atrial additional ablations were also counted.

As for the rhythm outcome, the AF recurrence rate was also examined for 2033 s AF ablation procedures performed in September. After excluding 308 patients without follow‐up data and 217 patients who underwent more than one left atrial additional ablation, 1508 patients were analyzed (the September cohort). To assess the rhythm outcome of the second ablation procedure, patients were divided into four groups according to ablation strategy, groups of each type of left atrial additional ablation, and groups of each type of left atrial linear ablation, as described above.

Rhythm outcomes after the second ablation procedure were followed for 12 months. Although Holter electrocardiography and 12‐lead electrocardiography were performed during follow‐up periods in accordance with a current guideline, methods of follow‐up were discrete in each institution discrete [7]. AF recurrence was defined as the presence of atrial tachyarrhythmias at 3 months after the procedure.

In the second ablation procedure and third or later ablation procedures, re‐PVI rates and types of left atrial additional ablation in each year were also evaluated.

### 2.4 Statistical Analysis

In this study, categorical data are presented as absolute values and percentages, and continuous data as means ± standard deviation or median (1st quartile–3rd quartile).

Tests for significance were conducted using the chi‐squared test or Cochran–Armitage test for categorical variables and one‐way analysis of variance or the Kruskal–Wallis test for continuous variables. Standardized mean difference analysis was also performed in order to evaluate the difference in patient characteristics between the September cohort and the non‐September cohort (patients other than those in the September cohort).

In Kaplan–Meier analysis, the log‐rank test was used to investigate the association between AF recurrence and ablation strategy. Cox proportional hazards regression analyses were also used to calculate the hazard ratio and 95% confidence interval of AF recurrence. All analyses were performed using commercial software (Stata version 17 [Stata Corp., College Station, TX, USA]).

## 3 Results

### 3.1 Patient Characteristics and Ablation Strategy

Patient characteristics stratified by ablation strategy in patients who underwent a second ablation procedure are shown in Table 1. There were significant differences in age, gender, prevalence of persistent AF (perAF), prevalence of structural heart disease, prevalence of drug‐refractory AF, and ablation system among groups. In addition, comparisons of patient characteristics in the second ablation stratified by cohort subtype are shown in Table 2. There were significant differences in the prevalence of ischemic heart disease, prevalence of valvular heart disease, prevalence of thromboembolism, and ablation system between the September and non‐September cohorts. In the standardized mean difference analysis, more patients underwent radiofrequency ablation in the non‐September cohort than in the September cohort. There was a significant difference in the rate of procedures with general anesthesia among four groups stratified by ablation strategy (neither re‐PVI nor left atrial additional ablation: 24.7%, re‐PVI: 20.5%, left atrial additional ablation: 35.3%, re‐PVI and left atrial additional ablation: 31.6%, *p* < 0.001). Conversely, there was no significant difference in the usage rate of contact force sensing catheter among four groups stratified by ablation strategy (neither re‐PVI nor left atrial additional ablation: 83.9%, re‐PVI: 79.0%, left atrial additional ablation: 81.6%, re‐PVI and left atrial additional ablation: 83.6%, *p* = 0.24).

**TABLE 1:** Patient characteristics in second ablation stratified by ablation strategy.

Variable	Ablation strategy				
	Neither re‐PVI nor additional ablation (*n* = 280)	Re‐PVI (*n* = 10 852)	Left atrial additional ablation (*n* = 4466)	Re‐PVI and left atrial additional ablation (*n* = 10 086)	*p*
Age, years	68 (60–74)	68 (60–74)	70 (63–76)	69 (62–75)	< 0.001
Female, *n* (%)	485 (37.9)	2895 (26.7)	1643 (36.8)	3064 (30.4)	< 0.001
Persistent AF, *n* (%)	356 (27.8)	3012 (27.8)	2408 (53.9)	4901 (48.6)	< 0.001
Body mass index, kg/m^2^	23.6 (21.5–25.7)	23.9 (21.8–26.4)	24.0 (21.7–26.5)	24.2 (21.9–26.6)	< 0.001
Structural heart disease, *n* (%)	136 (10.6)	1200 (11.1)	694 (15.5)	1581 (15.7)	< 0.001
Ischemic heart disease, *n* (%)	59 (4.6)	595 (5.5)	236 (5.3)	606 (6.0)	0.08
Old myocardial infarction, *n* (%)	17 (1.3)	217 (2.0)	96 (2.1)	242 (2.4)	0.04
Angina pectoris, *n* (%)	43 (3.4)	377 (3.5)	135 (3.0)	360 (3.6)	0.41
Valvular heart disease, *n* (%)	29 (2.3)	194 (1.8)	183 (4.1)	326 (3.2)	< 0.001
Other heart disease, *n* (%)	52 (4.1)	443 (4.1)	299 (6.7)	704 (7.0)	< 0.001
Dilated cardiomyopathy, *n* (%)	10 (0.8)	118 (1.1)	74 (1.7)	160 (1.6)	0.001
Hypertrophic cardiomyopathy, *n* (%)	32 (2.5)	199 (1.8)	150 (3.4)	365 (3.6)	< 0.001
Drug‐refractory AF, *n* (%)	501 (39.1)	4751 (43.8)	2019 (45.2)	4673 (46.3)	< 0.001
Thromboembolism, *n* (%)	63 (4.9)	659 (6.1)	306 (6.9)	665 (6.6)	0.03
Ablation system					
Radio frequency ablation, *n* (%)	1268 (99.1)	10 405 (95.9)	4442 (99.5)	9877 (97.9)	< 0.001
Cryoballoon ablation, *n* (%)	4 (0.3)	417 (3.8)	66 (1.5)	454 (4.5)	< 0.001
Hot balloon ablation, *n* (%)	0 (0.0)	92 (0.8)	1 (0.0)	93 (0.9)	< 0.001
Laser balloon ablation, *n* (%)	3 (0.2)	99 (0.9)	2 (0.0)	5 (0.0)	< 0.001

Abbreviations: AF, atrial fibrillation; PVI, pulmonary vein isolation.

**TABLE 2:** Comparisons of patient characteristics in second ablation stratified by cohort subtypes.

Variable	All (*n* = 26 684)	Non‐September cohort (*n* = 25 176)	September cohort (*n* = 1508)	*p* ^a^	SMD^a^
Age, years	69 (61–74)	69 (61–74)	69 (61–75)	0.80	−0.011
Female, *n* (%)	8087 (30.3)	7621 (30.3)	466 (30.9)	0.60	0.014
Persistent AF, *n* (%)	10 677 (40.0)	10 109 (40.2)	568 (37.7)	0.055	−0.051
Body mass index, kg/m^2^	24.0 (21.8–26.5)	24.0 (21.8–26.5)	23.9 (21.7–26.3)	0.16	−0.009
Structural heart disease, *n* (%)	3611 (13.5)	3388 (13.5)	223 (14.8)	0.14	0.038
Ischemic heart disease, *n* (%)	1496 (5.6)	1379 (5.5)	117 (7.8)	< 0.001	0.092
Old myocardial infarction, *n* (%)	572 (2.1)	534 (2.1)	38 (2.5)	0.30	0.026
Angina pectoris, *n* (%)	915 (3.4)	838 (3.3)	77 (5.1)	< 0.001	0.089
Valvular heart disease, *n* (%)	732 (2.7)	677 (2.7)	55 (3.6)	0.027	0.055
Other heart disease, *n* (%)	1498 (5.6)	1433 (5.7)	65 (4.3)	0.024	−0.063
Dilated cardiomyopathy, *n* (%)	362 (1.4)	342 (1.4)	20 (1.3)	0.92	−0.003
Hypertrophic cardiomyopathy, *n* (%)	746 (2.8)	713 (2.8)	33 (2.2)	0.14	−0.041
Drug‐refractory AF, *n* (%)	11 944 (44.8)	11 266 (44.7)	678 (45.0)	0.87	0.004
Heart failure, *n* (%)	—	—	231 (15.3)	—	
Hypertension, *n* (%)	—	—	702 (46.6)	—	
Diabetes mellitus, *n* (%)	—	—	215 (14.3)	—	
Thromboembolism, *n* (%)	1693 (6.3)	1617 (6.4)	76 (5.0)	0.032	−0.06
Thyroid dysfunction, *n* (%)	—	—	40 (2.7)	—	
COPD, *n* (%)	—	—	25 (1.7)	—	
Sleep apnea syndrome, *n* (%)	—	—	81 (5.4)	—	
Creatinine, mg/dL	—	—	0.9 (0.7–1.0)	—	
Left ventricular ejection fraction, %	—	—	63 (58–68)	—	
Left atrial diameter, mm	—	—	40 (36–45)	—	
Pre‐procedural cardiac imaging					
Computed tomography, *n* (%)	—	—	1270 (84.4)	—	
Magnetic resonance imaging, *n* (%)	—	—	15 (1.0)	—	
TEE, *n* (%)	—	—	607 (40.4)	—	
General anesthesia, *n* (%)			394 (26.8)	—	
Ablation system					
Radio frequency ablation, *n* (%)	25 992 (97.4)	24 554 (97.5)	1438 (95.4)	< 0.001	−0.117
Contact force sensing catheter, *n* (%)			1158 (81.3)	—	
Cryoballoon ablation, *n* (%)	941 (3.5)	901 (3.6)	40 (2.7)	0.058	−0.053
Hot balloon ablation, *n* (%)	186 (0.7)	163 (0.6)	23 (1.5)	< 0.001	0.085
Laser balloon ablation, *n* (%)	109 (0.4)	98 (0.4)	11 (0.7)	0.044	0.046

Abbreviations: AF, atrial fibrillation; COPD, chronic obstructive pulmonary disease; SMD, standardized mean difference; TEE, transesophageal echocardiography.

^a^ Comparison between the September cohort and the non‐September cohort.

**FIGURE 2:**
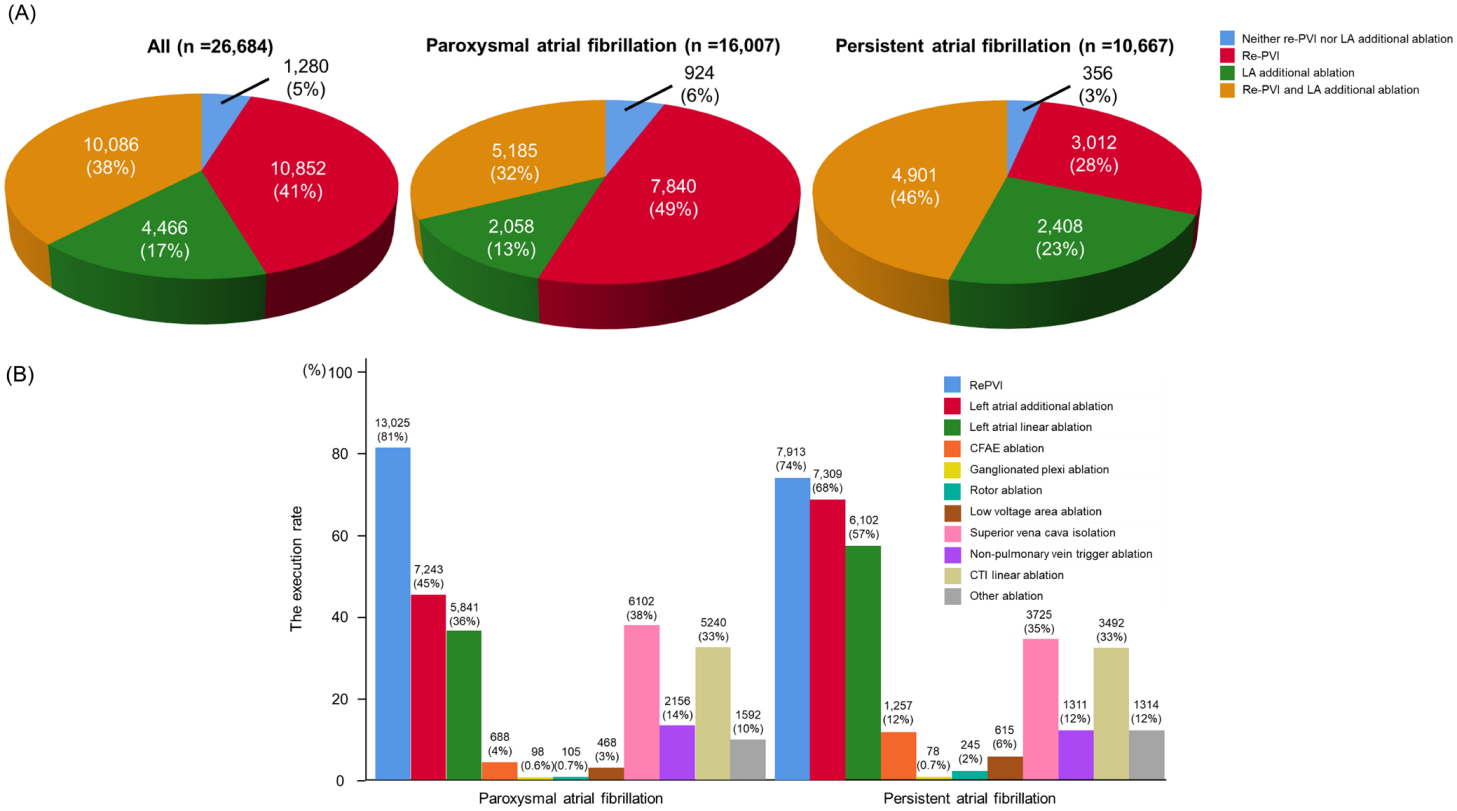
The execution rate stratified by ablation strategy. (A) In paroxysmal atrial fibrillation, 49% of patients underwent re‐PVI only, conversely, both of re‐PVI and left atrial additional ablation were performed 46% of patients in persistent atrial fibrillation. (B) Re‐PVI was performed for 78% of patients. The execution rate of left atrial additional ablation in persistent atrial fibrillation was higher than that in paroxysmal atrial fibrillation. CFAE, complex fractionated atrial electrogram; CTI, cavo‐tricuspid isthmus; LA, left atrial; PVI, pulmonary vein isolation.

The execution rate in four groups stratified by ablation strategy is shown in Figure 2A. In pAF, 49% of the patients underwent re‐PVI only, whereas in perAF, both of re‐PVI and left atrial additional ablation were performed in 46% of patients. The execution rate of each ablation strategy is also shown in Figure 2B. Overall, 20 938 (78%) patients underwent re‐PVI and 14 552 (55%) patients underwent left atrial additional ablation.

### 3.2 Annual Trend in Re‐PVI and Left Atrial Additional Ablation in Second Ablation Procedure

Annual trend in re‐PVI and left atrial additional ablation in second ablation procedure is shown in Figure S1. Re‐PVI rate showed an annual tendency to decrease over time. To the contrary, the left atrial additional ablation rate showed an annual tendency to increase over time.

Subanalysis of left atrial linear ablation in the second ablation procedure is shown in Figure S2. In left atrial linear ablation, left atrial posterior wall isolation showed an annual tendency to increase, while roof + MI showed an annual tendency to decrease.

### 3.3 Safety Outcome in Second Ablation Procedure

The overall complication rate in the second ablation procedure was 2.3% (pAF: 2.4%, perAF: 2.2%). As for the individually assessed complications in the second ablation procedure, the cardiac tamponade/effusion rate was 0.6% (pAF: 0.7%, perAF: 0.4%); the thromboembolism rate was 0.1% (pAF: 0.1%, perAF: 0.1%), and the esophageal complication rate was 0.1% (pAF: 0.1%, perAF: 0.1%). The majority of complications were other than individually assessed complications (1.5% [pAF: 1.4%, perAF: 1.6%]).

**FIGURE 3:**
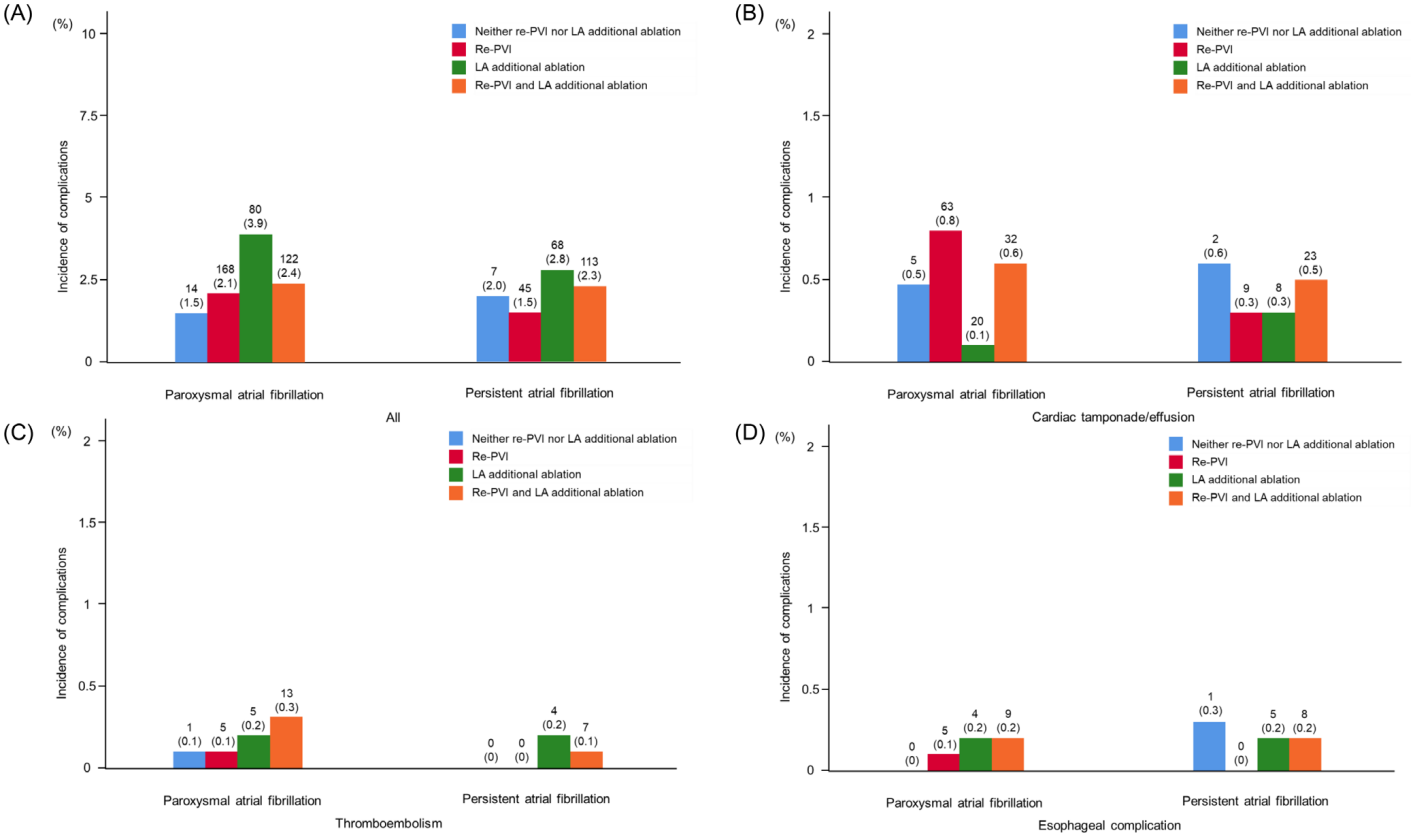
Complication rate stratified by ablation strategy in second ablation procedure. (A) All complications; (B) cardiac tamponade/effusion; (C) thromboembolism; (D) esophageal complications. LA, left atrial; PVI, pulmonary vein isolation.

The complication rates in four groups stratified by ablation strategy are shown in Figure 3. Complication rates ranged from 1.5% (pAF without re‐PVI nor left atrial additional ablation) to 3.9% (pAF with left atrial additional ablation only). Complication rates in the second ablation procedure stratified by ablation strategy are also shown in Figure S3. Among each ablation strategy, the lowest complication rate was 0% in perAF with GP ablation and the highest complication rate was 4.2% in pAF with CFAE ablation.

Additionally, subanalysis of complication rates stratified by the details of left atrial linear ablation is shown in Figure S4. Although complications occurred in 11.3% of pAF patients undergoing anterior mitral isthmus line, complication rates in patients with other strategies for left atrial linear ablation were less than 5%. Most complications with pAF patients who underwent anterior mitral isthmus line were other than individually assessed complications (9 [7.8%]).

**FIGURE 4:**
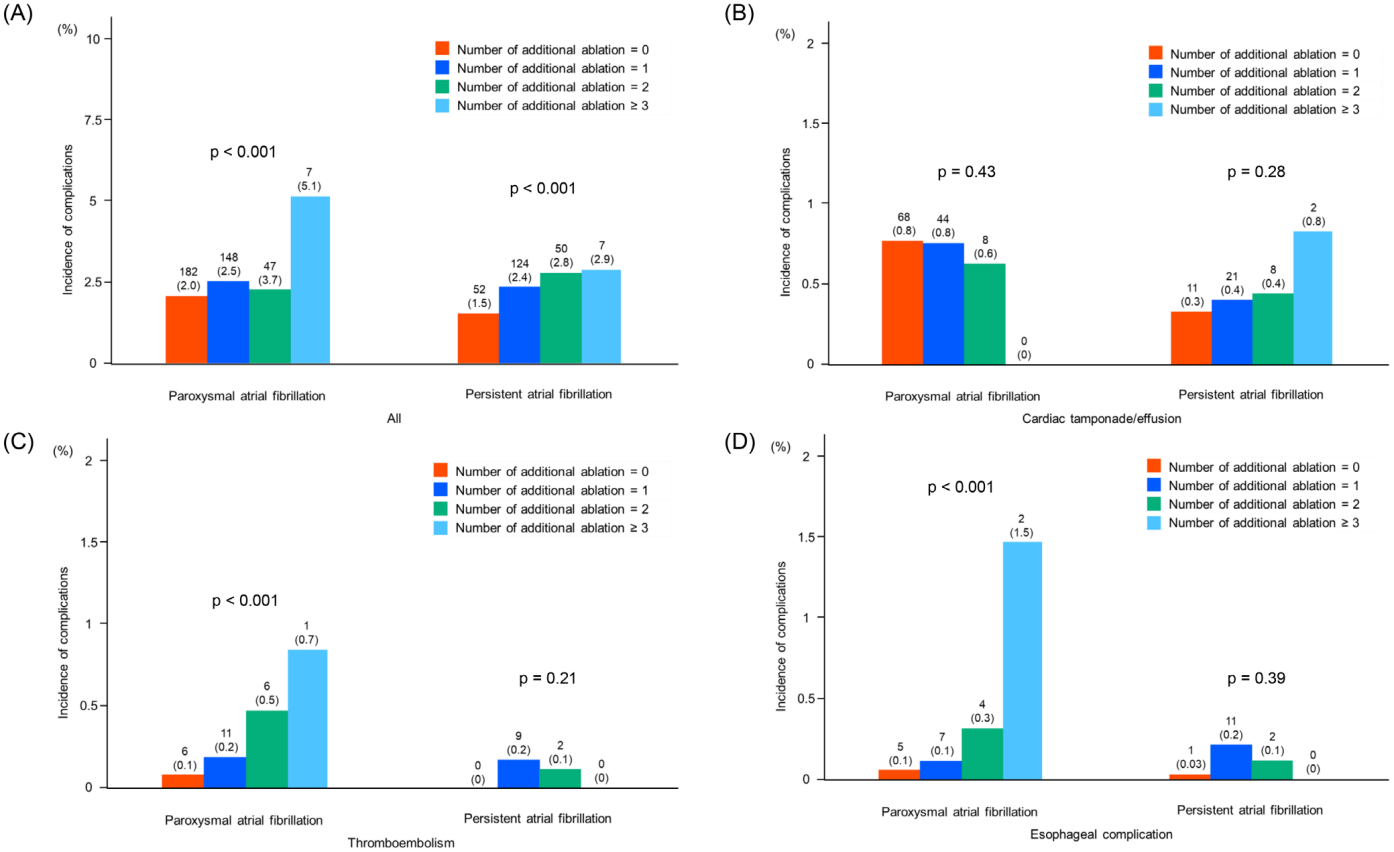
Complication rate stratified by the number of additional ablations in second ablation procedure. (A) All complications. As the number of additional ablations increased, the overall complication rate also increased. (B) Cardiac tamponade/effusion. There was no significant difference in cardiac tamponade/effusion rate by the number of additional ablations. (C) Thromboembolism. In patients with paroxysmal atrial fibrillation, as the number of additional ablations increased, the thromboembolism rate also significantly increased. (D) Esophageal complications. In patients with paroxysmal atrial fibrillation, as the number of additional ablations increased, the esophageal complication rate also significantly increased.

Complication rates stratified by the number of left atrial additional ablations are shown in Figure 4. As the number of left atrial additional ablations increased, the overall complication rate also significantly increased. In particular, as the number of left atrial additional ablations increased, the thromboembolism rate and esophageal complication rate in patients with pAF also significantly increased.

### 3.4 Efficacy Outcome in Second Ablation Procedure

**FIGURE 5:**
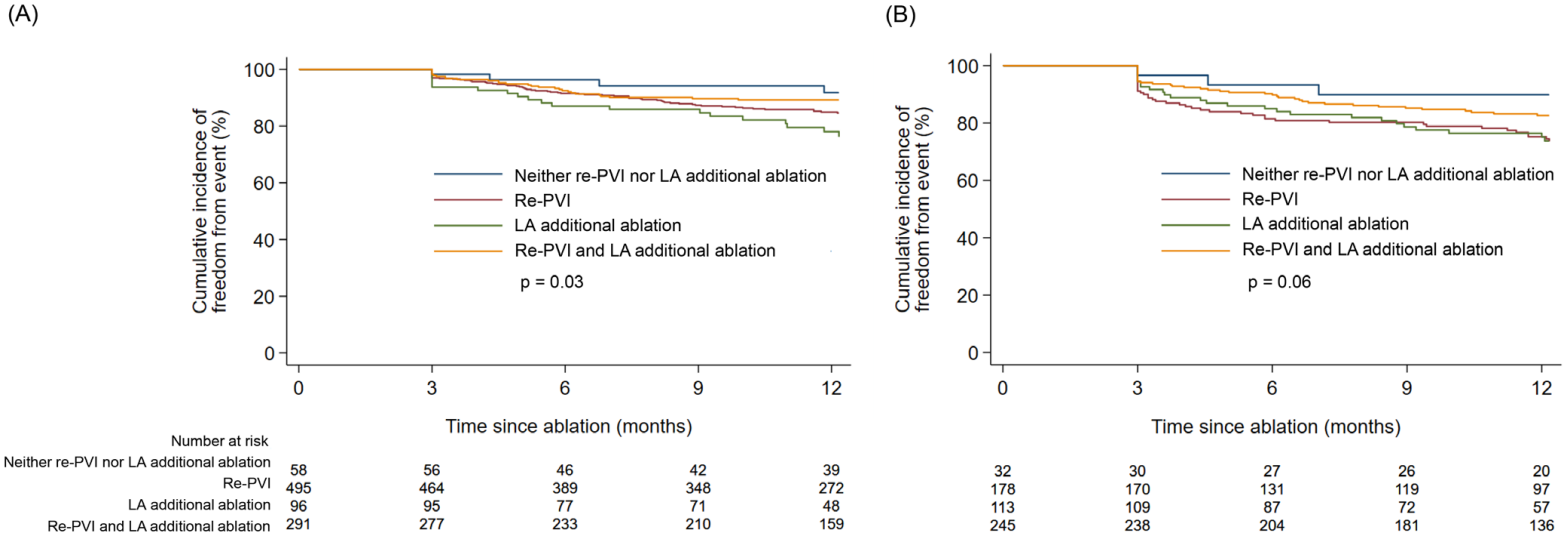
Freedom from AF recurrence following second ablation procedure. (A) In patients with pAF, freedom from AF recurrence was significantly lower in patients with additional ablation only than in those with neither re‐PVI nor additional ablation (hazard ratio, 3.0; 95% confidence interval, 1.04–8.9; *p* = 0.04 by Cox proportional hazards regression analysis). (B) In patients with perAF, there was no significant difference in AF recurrence rate among ablation strategies. AF, atrial fibrillation; LA, left atrial; pAF, paroxysmal atrial fibrillation; perAF, persistent atrial fibrillation; PVI, pulmonary vein isolation.

As for the rhythm outcome collected in the September cohort, the rate of freedom from AF recurrence was 87.6% for pAF and 80.6% for perAF during the 12 months following the second ablation procedure. Freedom from AF recurrence stratified by ablation strategy is shown in Figure 5. In pAF, freedom from AF recurrence was significantly lower in patients with left atrial additional ablation only than in those with neither re‐PVI nor left atrial additional ablation (hazard ratio, 3.0; 95% confidence interval, 1.04–8.9; *p* = 0.04 by Cox proportional hazards regression analysis). In the subanalysis, AF recurrence rates were evaluated with stratification by detailed left atrial additional ablation strategy. Additionally, because the majority of left atrial additional ablation strategies were left atrial linear ablation, AF recurrence rates were also evaluated with stratification by detailed left atrial linear ablation strategy. For both pAF and perAF, freedom from AF recurrence was similar among patients without left atrial additional ablation, those with left atrial linear ablation, and those with other types of left atrial additional ablation (Figure S5). There was also no significant difference in AF recurrence rate among left atrial linear ablation strategies, irrespective of AF type (Figure S6).

### 3.5 Ablation Procedure in Third or Later Ablation Procedure

Re‐PVI and left atrial additional ablation in the third or later ablation procedure are shown in Figure S7. Re‐PVI was required for 53% (pAF: 56%, perAF: 49%) of patients, and 75% (pAF: 70%, perAF: 82%) of patients underwent left atrial additional ablation. Although the re‐PVI rate showed an annual tendency to decrease over time in all AF and pAF, to the contrary, the left atrial additional ablation rate showed an annual tendency to increase over time in all AF and pAF.

Subanalysis of left atrial linear ablation in third or later ablation procedure is shown in Figure S8. In left atrial linear ablation for all AF and perAF, left atrial posterior wall isolation showed an annual tendency to increase over time, while LA roof + MI showed an annual tendency to decrease over time.

The overall complication rate in third or later ablation procedures was 2.3% (pAF: 2.3%, perAF: 2.4%). As for the individually assessed complications in third or later ablation procedures, the cardiac tamponade/effusion rate was 0.5% (pAF: 0.5%, perAF: 0.4%), the thromboembolism rate was 0.2% (pAF: 0.1%, perAF: 0.3%), and the esophageal complication rate was 0.1% (pAF: 0.1%, perAF: 0.1%).

## 4 Discussion

In this study, we investigate real‐world data for the usage, efficacy, and safety of re‐PVI and left atrial additional ablation in repeat ablation in Japan. Data were from 26 684 patients who underwent a second ablation procedure for AF and 6088 patients who underwent a third or later ablation procedure for AF, sourced from a multicenter, prospective, observational, nationwide cohort registry in Japan. Our main findings were as follows: (1) re‐PVI was performed for 20 938 (78%) patients, and 14 552 (55%) patients underwent left atrial additional ablation in the second ablation procedure; (2) almost half of the patients who underwent a third or later ablation procedure needed re‐PVI; (3) as the number of left atrial additional ablations increased, the overall complication rate in the second ablation procedure also increased; and (4) in the second ablation procedure, there was a significant difference in the AF recurrence rate among ablation strategies in patients with pAF; specifically, freedom from AF recurrence was significantly lower in patients with left atrial additional ablation only than in those with neither re‐PVI nor left atrial additional ablation.

To our knowledge, this is the first nationwide analysis to assess real‐world data in the usage, efficacy, and safety of repeat ablation.

### 4.1 Ablation Procedure in Second Ablation Procedure

Re‐PVI was performed in 78% of patients in this study. Other recent studies have reported that re‐PVI rates have been up to 65% [12, 13, 14], and the re‐PVI rate in this study is higher than these studies. Although previous studies were mainly conducted in high‐volume centers [12, 13, 14], this study included many cases performed by less experienced operators because J‐AB was a survey of almost all catheter ablation procedures performed in Japan, including both of high‐volume centers and low‐volume centers. Therefore, in real‐world clinical settings, re‐PVI is necessary in many cases of second ablation procedures.

Regarding left atrial additional ablation, a multicenter, prospective randomized‐controlled trial conducted in 2015 (STAR AF II) found that among patients with perAF, there was no reduction in the rate of AF recurrence between roof + MI plus PVI and PVI alone [3]. Based on the results of STAR AF II, roof + MI might show a tendency to decrease in this study period, and alternatively, left atrial posterior wall isolation might show an annual tendency to increase. Although the most recent randomized controlled trials have not demonstrated its efficacy in initial ablation [15], the efficacy of left atrial posterior wall isolation in patients undergoing repeat catheter ablation remains controversial. The reason for the result of this study may be that some studies prior to this study period have demonstrated a reduction in AF recurrence with left atrial posterior wall isolation [16, 17, 18].

### 4.2 Outcomes in Second Ablation Procedure

Regarding safety, the complication rate in the second ablation procedure was 2.2%–2.4%. Previous studies from the J‐AB registry reported complication rates in initial AF ablation of 2.5%–3%, and thus this rate appears similar in the initial and second ablation procedure [6, 19]. In the second ablation procedure, as the number of left atrial additional ablations increased, the complication rate also increased in both pAf and perAF. While left atrial additional ablation is performed to modify AF substrate [7], AF substrate develops due to aging and comorbidities, such as hypertension, diabetes mellitus, and heart failure, which are also risk factors for procedural complication [1, 20, 21, 22]. In addition, catheter ablation procedures with left atrial additional ablation require more procedural time than those with PVI only [3]. Hence, the complication rate in this study might increase with increasing the number of left atrial additional ablations. For example, complications occurred in 11.3% of patients with pAF who underwent an anterior mitral isthmus line, and this complication rate was more than twice that of patients with other strategies for left atrial linear ablation. Most of the complications in pAF patients undergoing anterior mitral isthmus line were other than individually assessed complications, which might mainly include access site hematoma [3, 8]. Therefore, the cause might be the long procedure time in anterior mitral isthmus line ablation [23]. In anterior mitral isthmus line ablation, procedure time is extended by ablating a long region [24, 25], and therefore, access site hematoma may increase by increasing the total heparin dose [26]. Additionally, patients who underwent anterior mitral isthmus line ablation might have arrhythmogenic substrates in the left anterior atrium caused by being elderly and underweight, which were also risk factors for procedural complications [27, 28, 29, 30]. In another example, the complication rate in patients with pAF undergoing CFAE ablation was also high in this study. CFAE also reflects age‐related electroanatomical remodeling, which is a common risk factor for procedural complications [30, 31, 32]. We consider it may be preferable for operators to avoid unnecessary left atrial additional ablation.

In terms of efficacy outcomes, in only pAF, freedom from AF recurrence was significantly lower in patients with left atrial additional ablation only than in those with neither re‐PVI nor left atrial additional ablation. The reason may be that there is a limitation to the effectiveness of left atrial additional ablation. Atrial remodeling is a diffuse process [33], and not all arrhythmogenicity can be modified by left atrial additional ablation. In addition, cases for which operators decided to perform left atrial additional ablation seemed to be difficult to maintain sinus rhythm. In the neither re‐PVI nor additional ablation group, ablation of non‐pulmonary vein foci, superior vena cava, or cavo‐tricuspid isthmus alone might have been considered to be sufficient.

### 4.3 Ablation Procedure in Third or Later Ablation Procedure

More than half of patients still needed re‐PVI in third or later ablation procedures. Although few reports of the PV reconnection rate in third or later ablation procedures have appeared, we found PV reconnection in a majority of patients with third or later ablation procedures. PV reconnection is the main cause of AF recurrence [34], and thus durable PVI in the initial and second ablation procedures appears important.

### 4.4 Clinical Implications

There are several clinical implications of this study. Because few studies about repeat ablation have appeared, left atrial additional ablations other than re‐PVI have been performed in individual institutions in the absence of a clinical consensus. Statistical data in repeat ablation are helpful in setting strategies for repeat ablation and in predicting efficacy and safety outcomes after repeat ablation. Additionally, the results of this study may be useful in developing future guidelines for AF ablation.

This study has also shown that complication rates increase as the number of left atrial additional ablations increases. Omission of unnecessary and empirical left atrial additional ablation can lead to a reduction in complications and savings of procedural costs.

### 4.5 Limitations

Some limitations of this study warrant mention. Initial catheter ablation data were not available in this study because repeat ablation data in the J‐AB data were not linked to the initial ablation. For example, the rate of atrial tachyarrhythmia recurrence following the initial ablation procedure was unknown. In addition, as is also seen among strategies in repeat ablation, strategies for left atrial additional ablation following initial PVI may differ among institutions. Further, we lacked data on radiofrequency settings during the procedure and endpoints for additional left atrial ablation, such as the success rate of bidirectional block in left atrial linear ablation or AF termination rate in CFAE ablation.

With regard to outcomes, methods of follow‐up were not consistent, albeit that we attempted to standardize these in accordance with a current guideline [7]. Furthermore, the study had an observational design and was conducted in a single country. Efficacy outcomes longer than 12 months and in patients who underwent catheter ablation other than September were not evaluated in this study. Finally, a degree of bias in the selection of left atrial additional ablation strategies and reported comorbidities may have been present. For example, each operator's indication for additional left atrial ablation strategies could be influenced by the progression of the AF substrate, which in turn could affect complication rates and AF recurrence.

## 5 Conclusions

In the second ablation procedure in Japan, re‐PVI was performed in 78% of patients, and both of re‐PVI and left atrial additional ablation were performed in 38% of patients. With regard to safety outcomes, the complication rate in the second ablation procedure was 2.3%, and as the number of left atrial additional ablations increased, the complication rate also increased. In the third or later ablation procedure, almost half of the patients required re‐PVI. In terms of rhythm outcomes, there was a significant difference in AF recurrence rates among ablation strategies in patients with pAF, which may be due not only to differences in procedural efficacy but also to differences in the underlying arrhythmogenic substrate.

## Ethics Statement

The protocol was approved by the Institutional Review Board of the National Cerebral and Cardiovascular Center (M28‐114‐7; approved December 2016), as well as by the institutional review boards of all participating hospitals. Registry and the Registration No. of the study/trial: The J‐AB registry is registered in the UMIN Clinical Trial Registry (UMIN 000028288) and ClinicalTrials.gov (NCT03729232). Animal Studies: Not applicable.

## Consent

Written informed consent for ablation and written informed consent or an opt‐out arrangement for participation in the study was obtained from all patients.

## Conflicts of Interest

Dr. Matsuda received honoraria from Daiichi Sankyo, Boehringer Ingelheim, Bayer, Medtronic, Boston Scientific Japan, Japan Lifeline, Asahi Kasei ZOLL Medical, Synaptic Medical Japan, Biotronik. Dr. Matsuda has also received a scholarship from the Japanese Heart Rhythm Society, Abbott and Nihon Kohden outside the submitted work. Dr. Masuda received honoraria from Daiichi Sankyo, Medtronic, Boston Scientific, and Johnson and Johnson. Dr. Inoue has received honoraria from Daiichi Sankyo, Nippon Boehringer Ingelheim, Bristol‐Meyers Squibb, Bayer Yakuhin, Johnson and Johnson KK., Boston Scientific Japan, and Medtronic Japan. Dr. Miyamoto has received funding/grants from Medtronic, Biosense Webster, and Abbott and speakers' bureaus from Medtronic, Biosense Webster, Abbott, Daiichi Sankyo, Nippon Boehringer Ingelheim, Bristol‐Meyers Squibb, Pfizer, Bayer Yakuhin, BEG company outside the submitted work. Dr. Satomi has received honoraria from Medtronic Japan, Abbott Medical Japan and Japan Lifeline. Dr. Kusano has received honoraria from Daiichi Sankyo, Nippon Boehringer Ingelheim, Biotronik Japan, Bayer Yakuhin, Pfizer Japan, and Medtronic Japan, and research grants from Medtronic Japan, HITACHI, Biotronic Japan, Mebix, and JSR. Dr. Yamane has received honoraria from Daiichi Sankyo, Medtronic Japan, and BEG Company Ltd.; and research grants from Nippon Boehringer Ingelheim. Dr. Shimizu has received honoraria from Daiichi Sankyo, Nippon Boehringer Ingelheim, Bristol‐Meyers Squibb, Bayer Yakuhin, Pfizer Japan, Novartis Pharma, Johnson and Johnson KK., and Boston Scientific Japan, and Medtronic Japan and research grants from Daiichi Sankyo and Nippon Boehringer Ingelheim.

## Supporting information


**Figure S1:** Annual tendency of re‐PVI and additional ablation in second ablation procedure. (A) Re‐PVI rate showed an annual tendency to decrease. In contrast, additional ablation rate showed an annual tendency to increase. Left atrial linear ablation, CFAE ablation, GP ablation, and rotor ablation showed an annual tendency to increase, while low voltage area ablation showed an annual tendency to decrease. (B) In pAF, re‐PVI rate showed an annual tendency to decrease. In contrast, additional ablation rate showed an annual tendency to increase. Left atrial linear ablation and GP ablation showed an annual tendency to increase, and low voltage area ablation showed an annual tendency to decrease. (C) In perAF, re‐PVI rate showed an annual tendency to decrease. In contrast, additional ablation rate showed an annual tendency to increase. Left atrial linear ablation, CFAE ablation, GP ablation, and rotor ablation showed an annual tendency to increase, and low voltage area ablation showed an annual tendency to decrease. PVI: pulmonary vein isolation, pAF: paroxysmal atrial fibrillation, perAF: persistent atrial fibrillation, LA: left atrial, CFAE: complex fractionated atrial electrogram, GP: ganglionated plexi, LVA: low voltage area.

**Figure S2:** Annual tendency of LA linear ablation in second ablation procedure. (A) LA posterior wall isolation showed an annual tendency to increase. In contrast, LA roof + MI showed an annual tendency to decrease. (B) In pAF, LA posterior wall isolation showed an annual tendency to increase. In contrast, LA roof + MI showed an annual tendency to decrease. (C) In perAF, LA posterior wall isolation showed an annual tendency to increase. In contrast, LA roof + MI showed an annual tendency to decrease. LA: left atrial, MI: mitral isthmus, pAF: paroxysmal atrial fibrillation, perAF: persistent atrial fibrillation, PW: posterior wall.

**Figure S3:** Complication rates in second ablation procedure stratified by ablation strategy. (A) All complications. (B) Cardiac tamponade/effusion. (C) Thromboembolism. (D) Esophageal complications. PVI: pulmonary vein isolation, CFAE: complex fractionated atrial electrogram, CTI: cavo‐tricuspid isthmus.

**Figure S4:** Complication rate stratified by details of left atrial linear ablation in second ablation procedure. (A) All complications. (B) Cardiac tamponade/effusion. (C) Thromboembolism. (D) Esophageal complications.

**Figure S5:** Freedom from AF recurrence stratified by the details of additional ablation. (A) In patients with pAF and other than additional ablation only, there was no significant difference in AF recurrence rate among groups. (B) In patients with pAF and additional ablation only, there was no significant difference in AF recurrence rate between patients with LA linear ablation and patients with other LA ablation. (C) In patients with perAF and other than additional ablation only, there was no significant difference in AF recurrence rate among groups. (D) In patients with perAF and additional ablation only, there was no significant difference in AF recurrence rate between patients with LA linear ablation and patients with other LA ablation. AF: atrial fibrillation, pAF: paroxysmal atrial fibrillation, perAF: persistent atrial fibrillation, PVI: pulmonary vein isolation, LA: left atrial.

**Figure S6:** Freedom from AF recurrence stratified by details of LA linear ablation. (A) In patients with pAF and other than LA linear ablation only, there was no significant difference in AF recurrence rate among groups. (B) In patients with pAF and LA linear ablation only, there was no significant difference in AF recurrence rate between patients with LA posterior wall isolation and patients with other LA linear ablation. (C) In patients with perAF and other than LA linear ablation only, there was no significant difference in AF recurrence rate among groups. (D) In patients with perAF and LA linear ablation only, there was no significant difference in AF recurrence rate between patients with LA posterior wall isolation and patients with other LA linear ablation. AF: atrial fibrillation, pAF: paroxysmal atrial fibrillation, perAF: persistent atrial fibrillation, LA: left atrial.

**Figure S7:** Annual tendency of re‐PVI and additional ablation in third or later ablation procedure. (A) Re‐PVI rate showed an annual tendency to decrease. In contrast, additional ablation rate showed an annual tendency to increase. Left atrial linear ablation and rotor ablation showed an annual tendency to increase, and low voltage area ablation showed an annual tendency to decrease. (B) In pAF, re‐PVI rate showed an annual tendency to decrease. In contrast, additional ablation rate showed an annual tendency to increase. Left atrial linear ablation and rotor ablation showed an annual tendency to increase, and low voltage area ablation showed an annual tendency to decrease. (C) In perAF, there was no annual tendency in re‐PVI rate or additional ablation rate. Rotor ablation showed an annual tendency to increase, while low voltage area ablation showed an annual tendency to decrease. PVI: pulmonary vein isolation, pAF: paroxysmal atrial fibrillation, perAF: persistent atrial fibrillation, LA: left atrial, CFAE: complex fractionated atrial electrogram, GP: ganglionated plexi, LVA: low voltage area.

**Figure S8:** Annual tendency of LA linear ablation in third or later ablation procedure. (A) LA posterior wall isolation showed an annual tendency to increase. In contrast, roof + MI showed an annual tendency to decrease over time. (B) In pAF, there was no annual tendency to increase or decrease for any strategy. (C) In perAF, LA posterior wall isolation showed an annual tendency to increase. In contrast, roof + MI showed an annual tendency to decrease over time. LA: left atrial, MI: mitral isthmus, pAF: paroxysmal atrial fibrillation, perAF: persistent atrial fibrillation, PW: posterior wall.

## Data Availability

Data in this study will not be shared.
