# Supplementary figures and images for: Real‐World Outcomes of Repeat Ablation Strategies for Atrial Fibrillation: Insights From the Japanese Catheter Ablation Registry
Source: J Arrhythm. 2025 Sep 22;41(5):e70200. doi: 10.1002/joa3.70200 (PMC12454675; doi:10.1002/joa3.70200)

(A) All

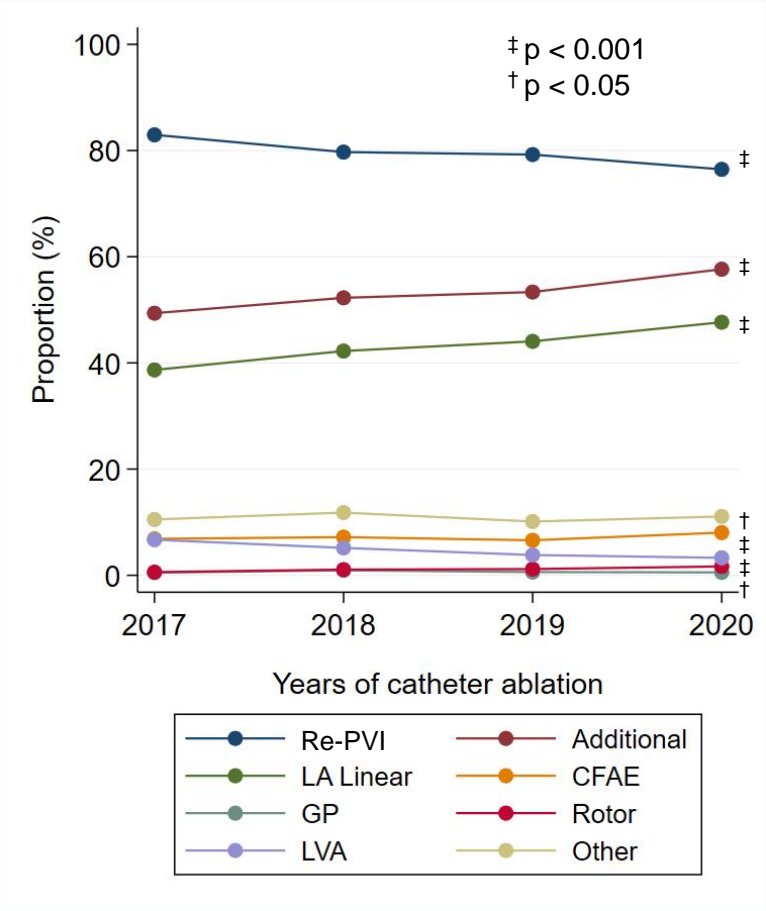

(B) pAF

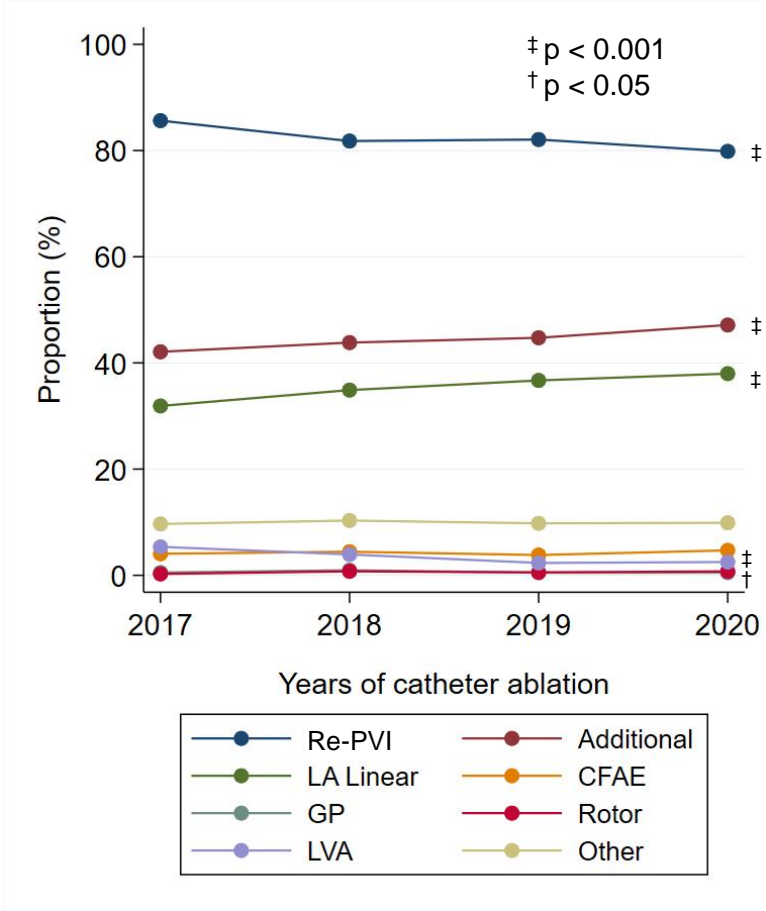

(C) perAF

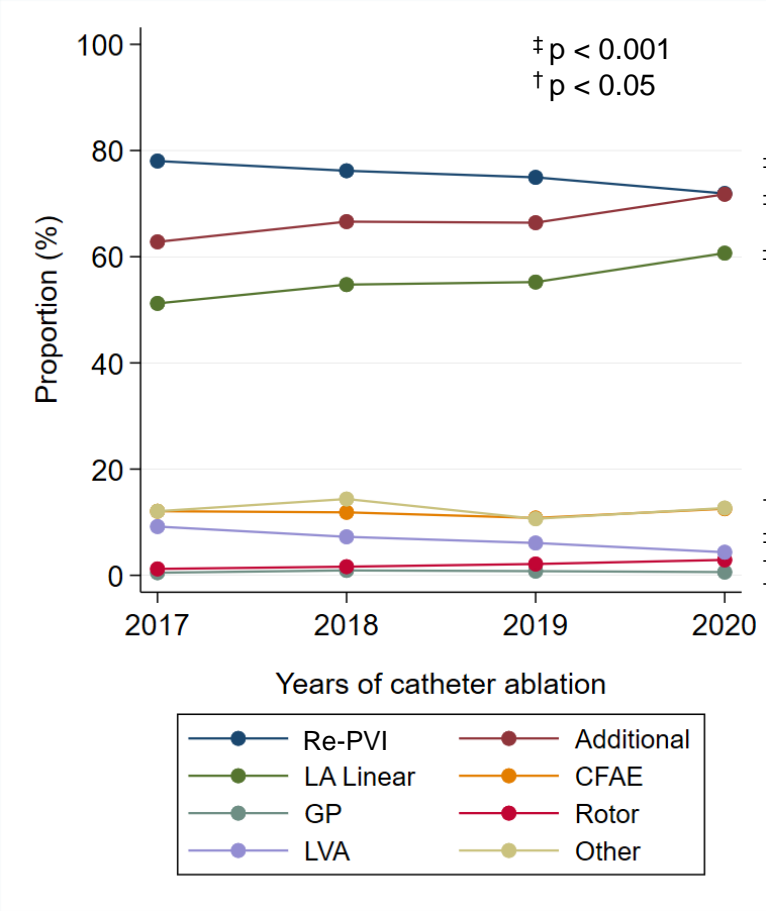

Supplement: Supplementary file 1 — Figure S1: Annual tendency of re‐PVI and additional ablation in second ablation procedure. (A) Re‐PVI rate showed an annual tendency to decrease. In contrast, additional ablation rate showed an annual tendency to increase. Left atrial linear ablation, CFAE ablation, GP ablation, and rotor ablation showed an annual tendency to increase, while low voltage area ablation showed an annual tendency to decrease. (B) In pAF, re‐PVI rate showed an annual tendency to decrease. In contrast, additional ablation rate showed an annual tendency to increase. Left atrial linear ablation and GP ablation showed an annual tendency to increase, and low voltage area ablation showed an annual tendency to decrease. (C) In perAF, re‐PVI rate showed an annual tendency to decrease. In contrast, additional ablation rate showed an annual tendency to increase. Left atrial linear ablation, CFAE ablation, GP ablation, and rotor ablation showed an annual tendency to increase, and low voltage area ablation showed an annual tendency to decrease. PVI: pulmonary vein isolation, pAF: paroxysmal atrial fibrillation, perAF: persistent atrial fibrillation, LA: left atrial, CFAE: complex fractionated atrial electrogram, GP: ganglionated plexi, LVA: low voltage area. [file JOA3-41-e70200-s001.pdf]

(A) All

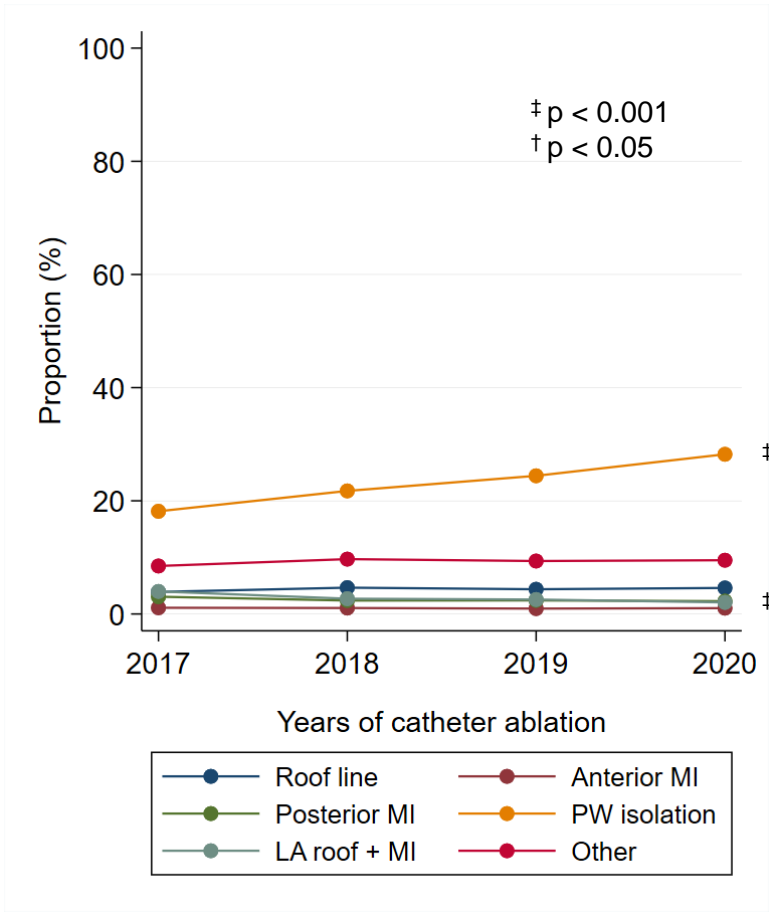

(B) pAF

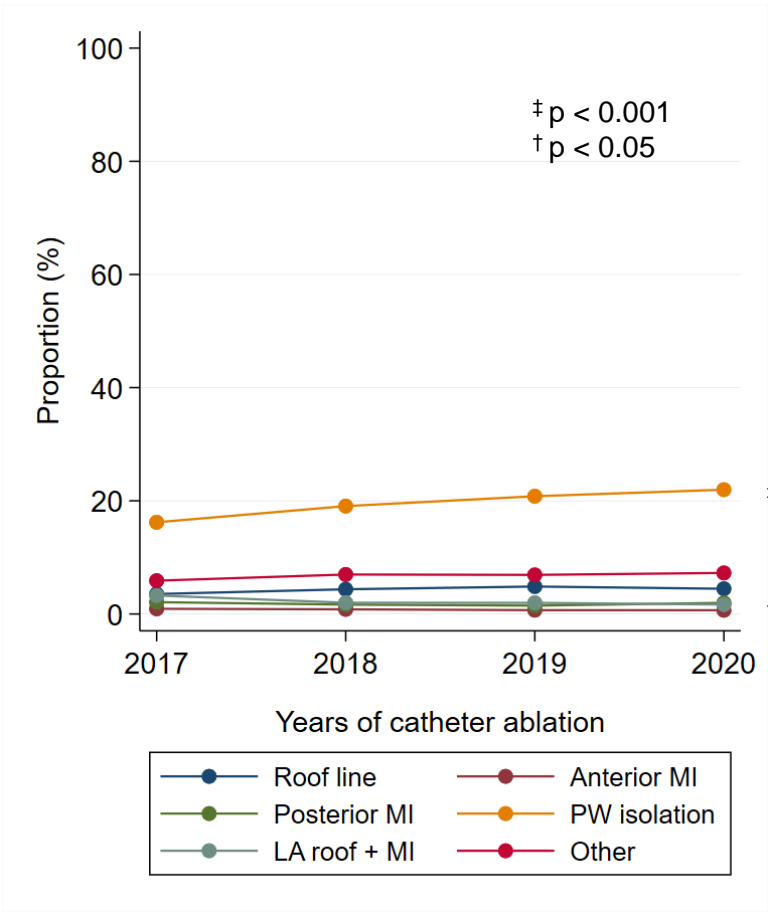

(C) perAF

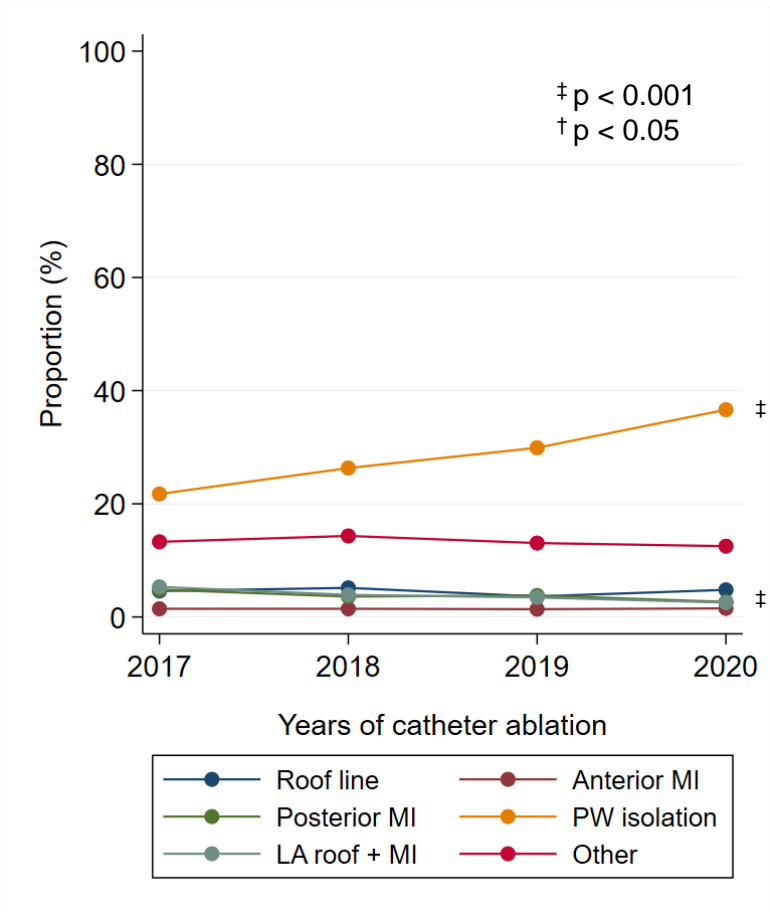

Supplement: Supplementary file 2 — Figure S2: Annual tendency of LA linear ablation in second ablation procedure. (A) LA posterior wall isolation showed an annual tendency to increase. In contrast, LA roof + MI showed an annual tendency to decrease. (B) In pAF, LA posterior wall isolation showed an annual tendency to increase. In contrast, LA roof + MI showed an annual tendency to decrease. (C) In perAF, LA posterior wall isolation showed an annual tendency to increase. In contrast, LA roof + MI showed an annual tendency to decrease. LA: left atrial, MI: mitral isthmus, pAF: paroxysmal atrial fibrillation, perAF: persistent atrial fibrillation, PW: posterior wall. [file JOA3-41-e70200-s007.pdf]

(A)

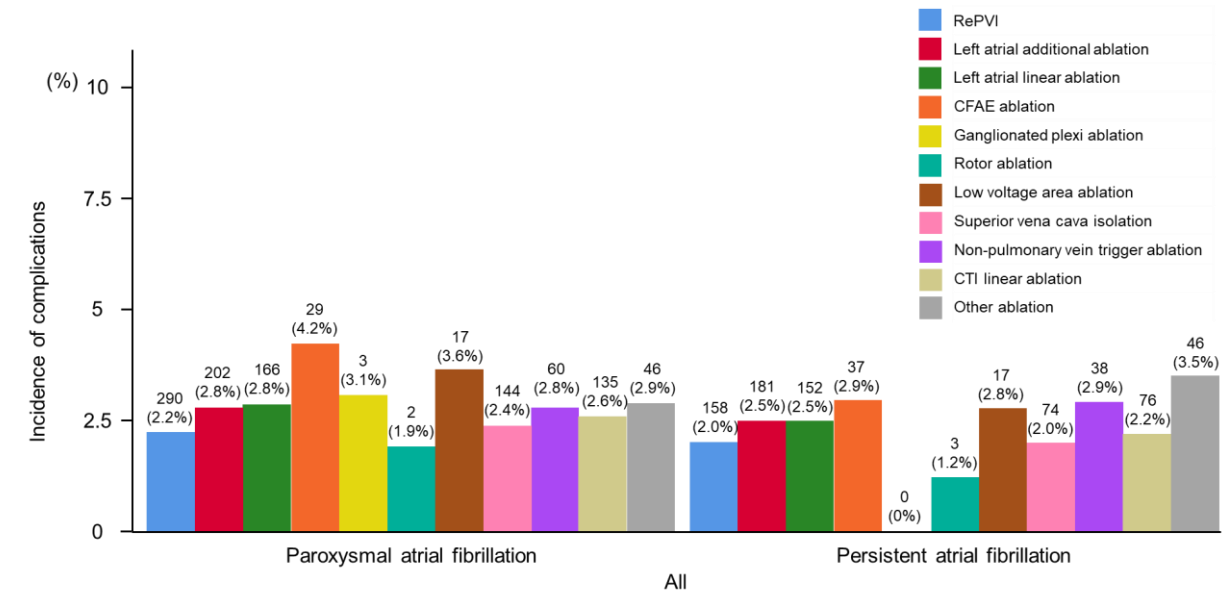

(B)

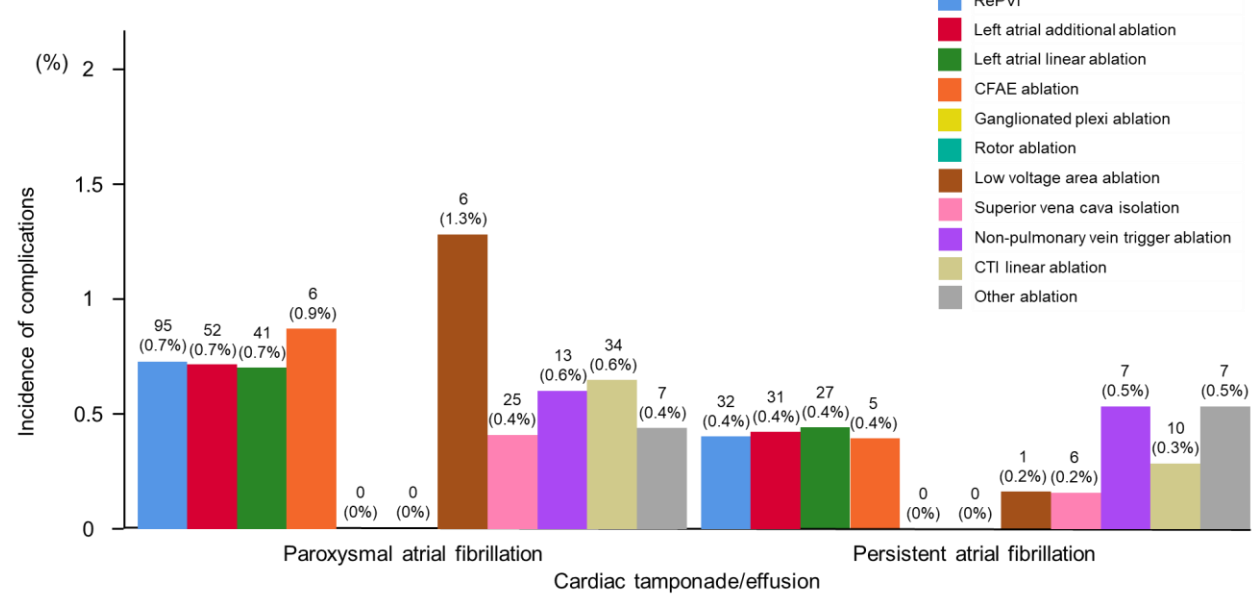

(C)

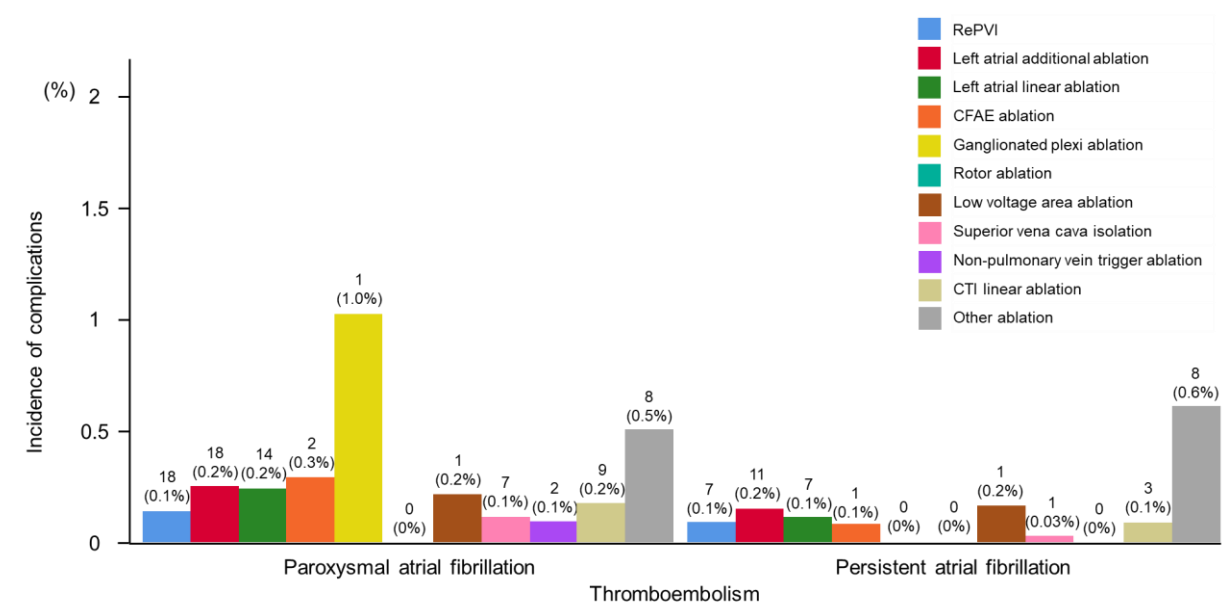

(D)

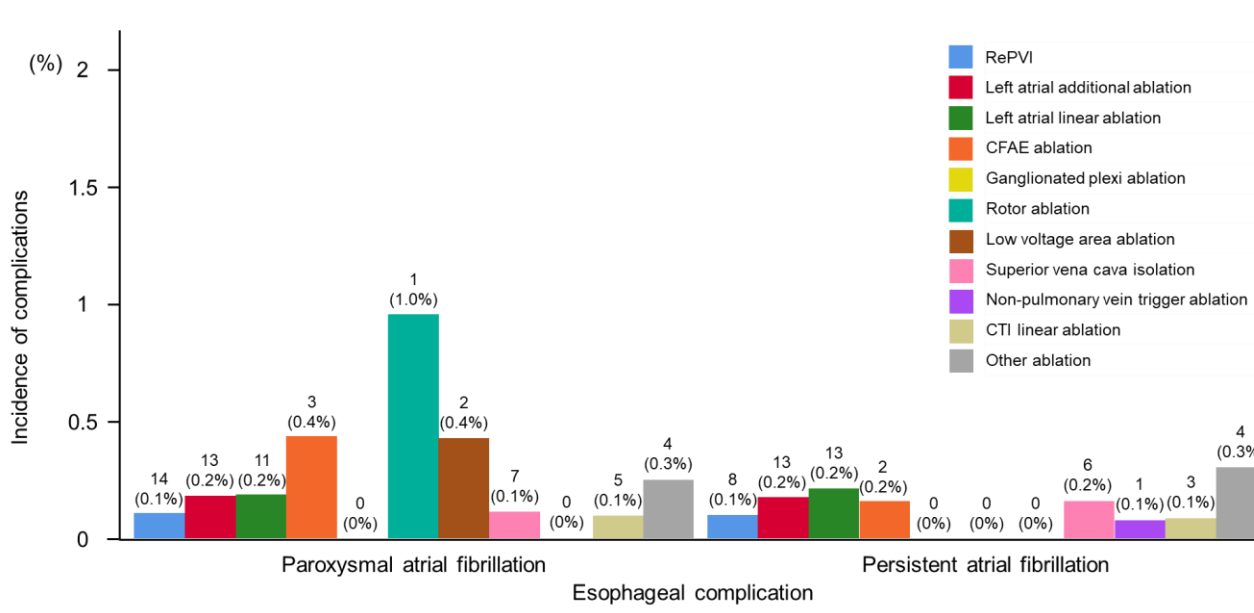

Supplement: Supplementary file 3 — Figure S3: Complication rates in second ablation procedure stratified by ablation strategy. (A) All complications. (B) Cardiac tamponade/effusion. (C) Thromboembolism. (D) Esophageal complications. PVI: pulmonary vein isolation, CFAE: complex fractionated atrial electrogram, CTI: cavo‐tricuspid isthmus. [file JOA3-41-e70200-s006.pdf]

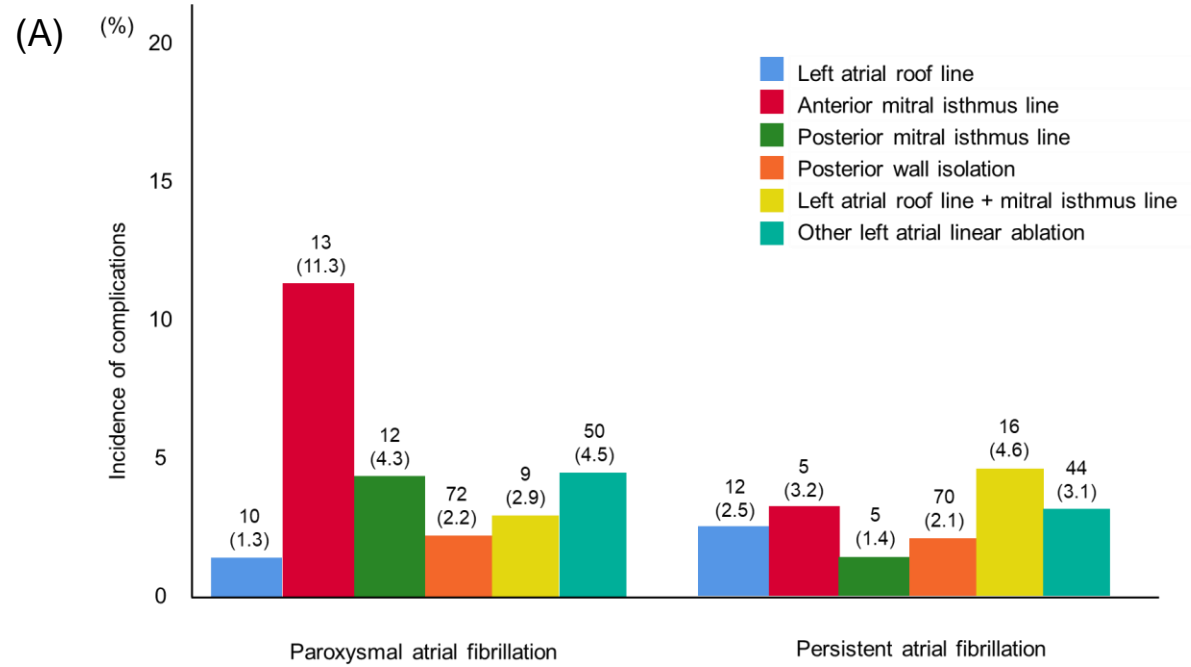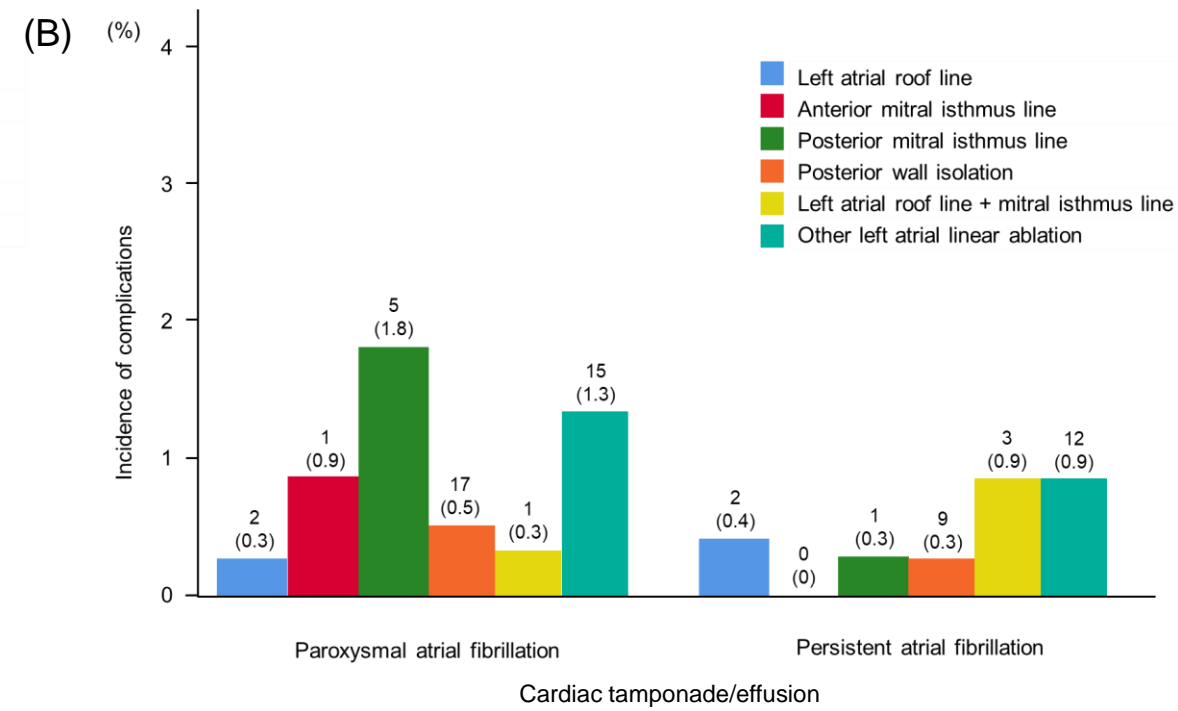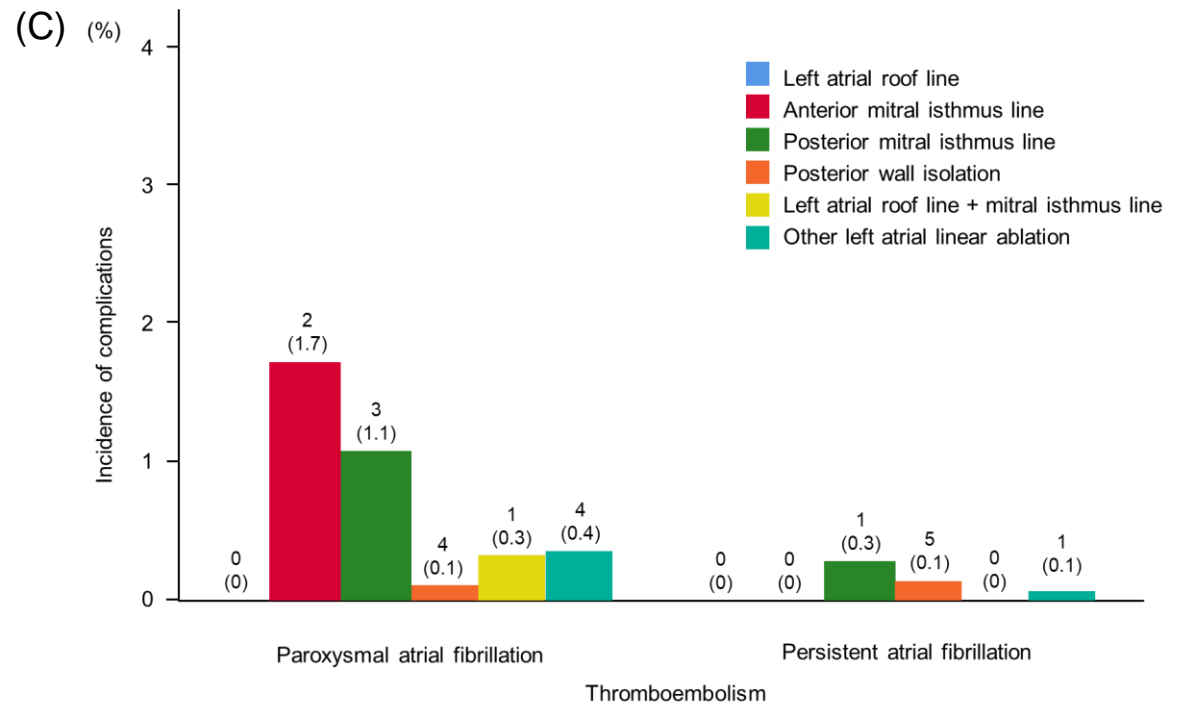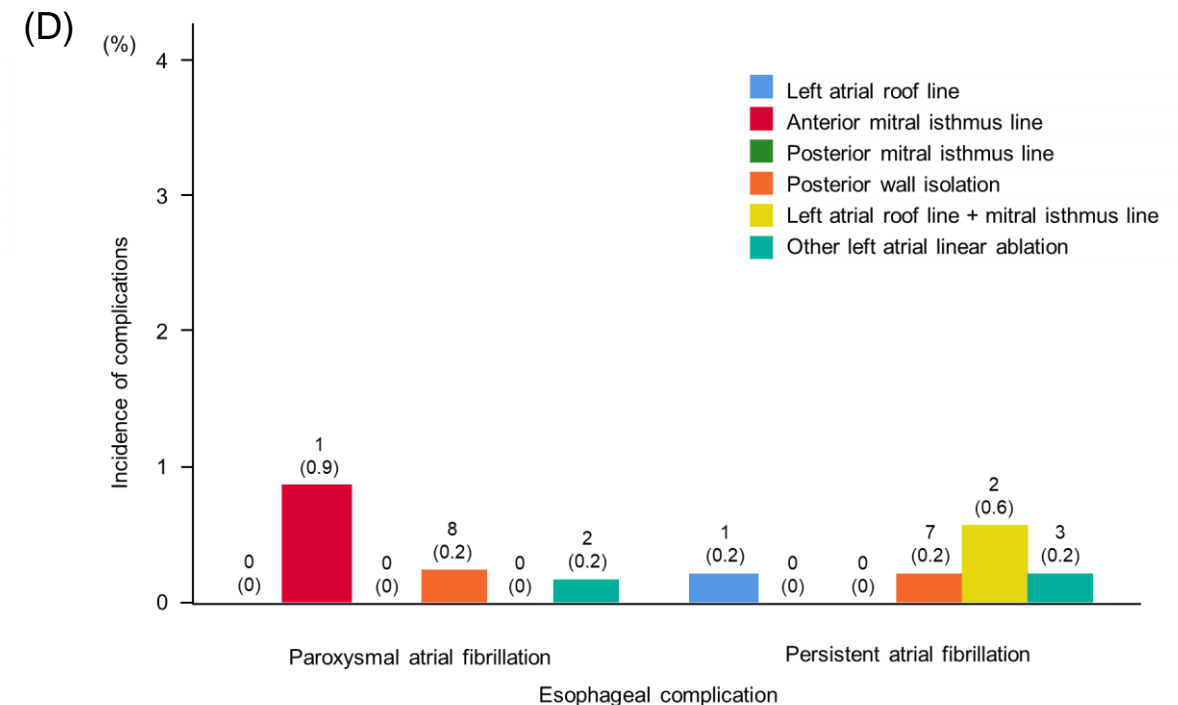

Supplement: Supplementary file 4 — Figure S4: Complication rate stratified by details of left atrial linear ablation in second ablation procedure. (A) All complications. (B) Cardiac tamponade/effusion. (C) Thromboembolism. (D) Esophageal complications. [file JOA3-41-e70200-s004.pdf]

(A) All

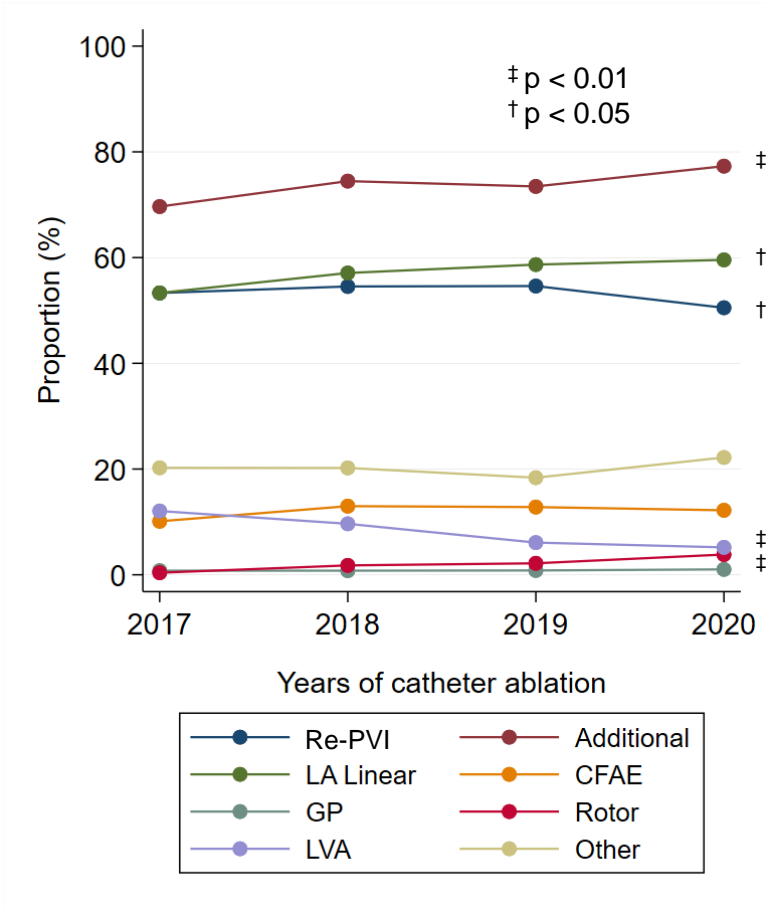

(B) pAF

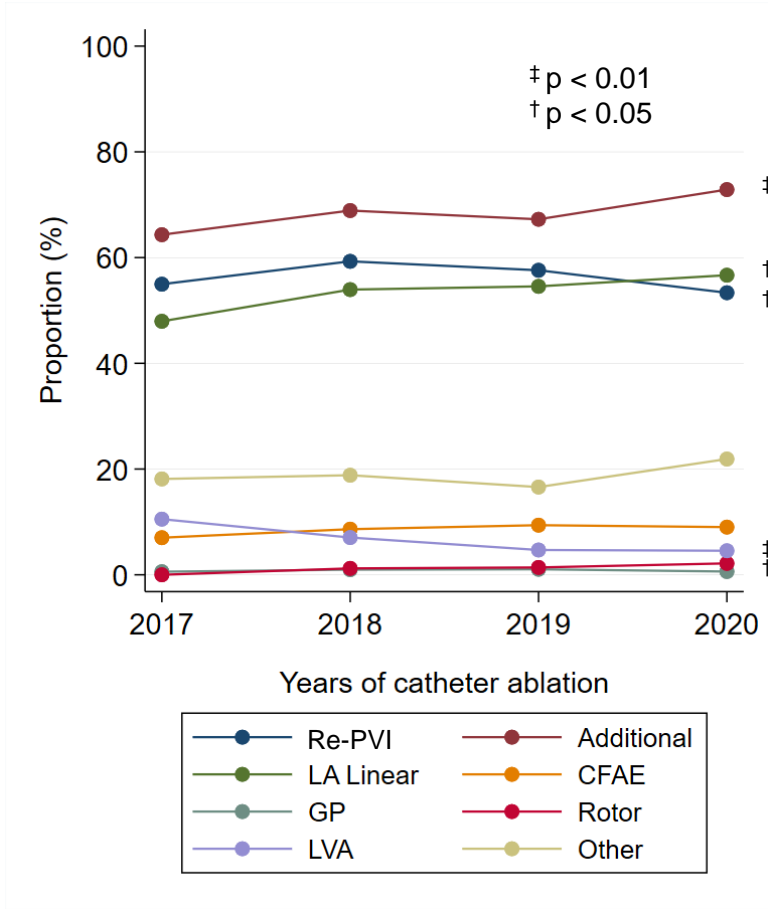

(C) perAF

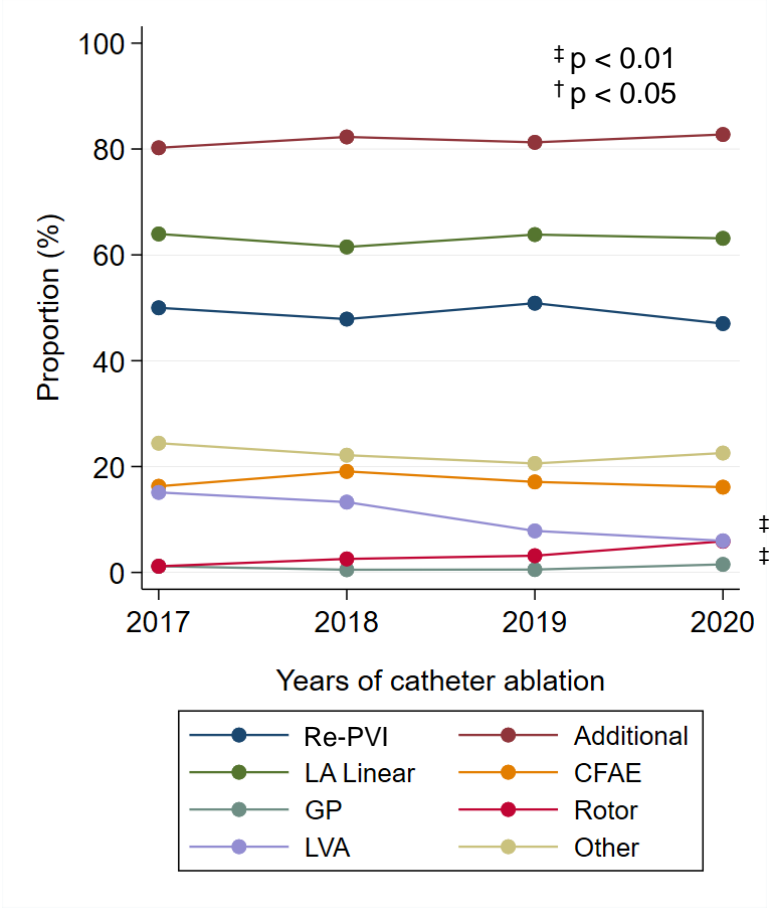

Supplement: Supplementary file 7 — Figure S7: Annual tendency of re‐PVI and additional ablation in third or later ablation procedure. (A) Re‐PVI rate showed an annual tendency to decrease. In contrast, additional ablation rate showed an annual tendency to increase. Left atrial linear ablation and rotor ablation showed an annual tendency to increase, and low voltage area ablation showed an annual tendency to decrease. (B) In pAF, re‐PVI rate showed an annual tendency to decrease. In contrast, additional ablation rate showed an annual tendency to increase. Left atrial linear ablation and rotor ablation showed an annual tendency to increase, and low voltage area ablation showed an annual tendency to decrease. (C) In perAF, there was no annual tendency in re‐PVI rate or additional ablation rate. Rotor ablation showed an annual tendency to increase, while low voltage area ablation showed an annual tendency to decrease. PVI: pulmonary vein isolation, pAF: paroxysmal atrial fibrillation, perAF: persistent atrial fibrillation, LA: left atrial, CFAE: complex fractionated atrial electrogram, GP: ganglionated plexi, LVA: low voltage area. [file JOA3-41-e70200-s002.pdf]

(A) All

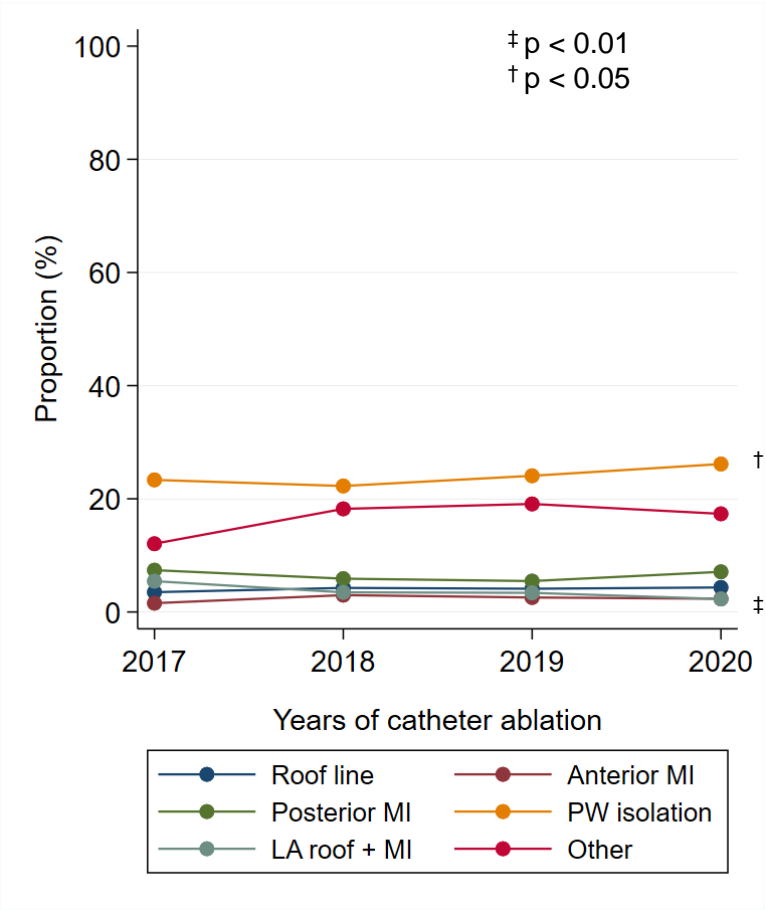

(B) pAF

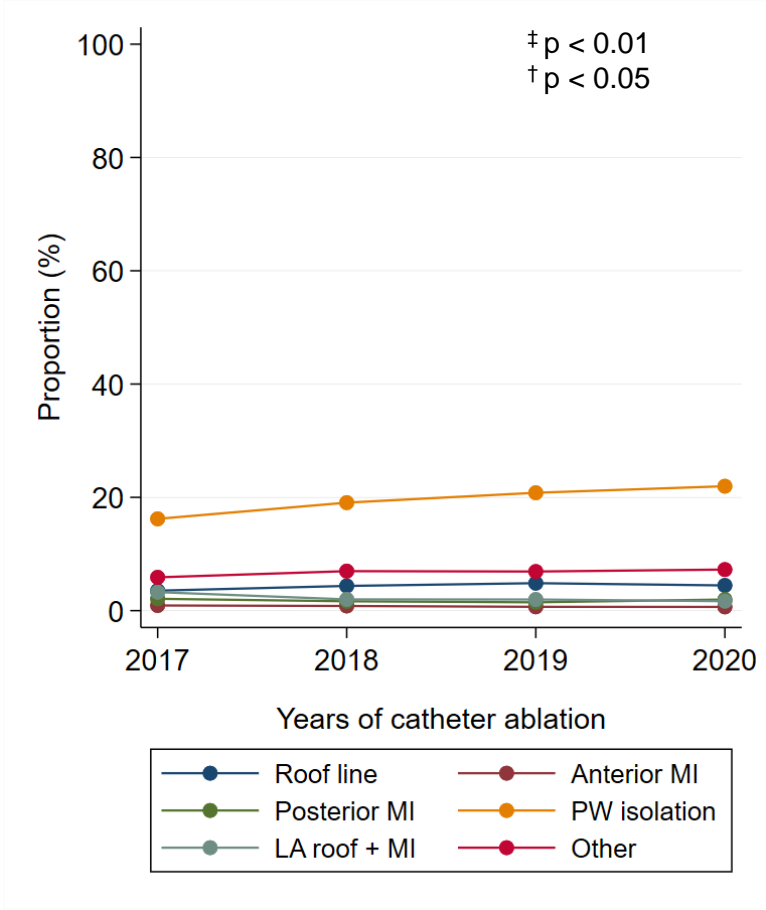

(C) perAF

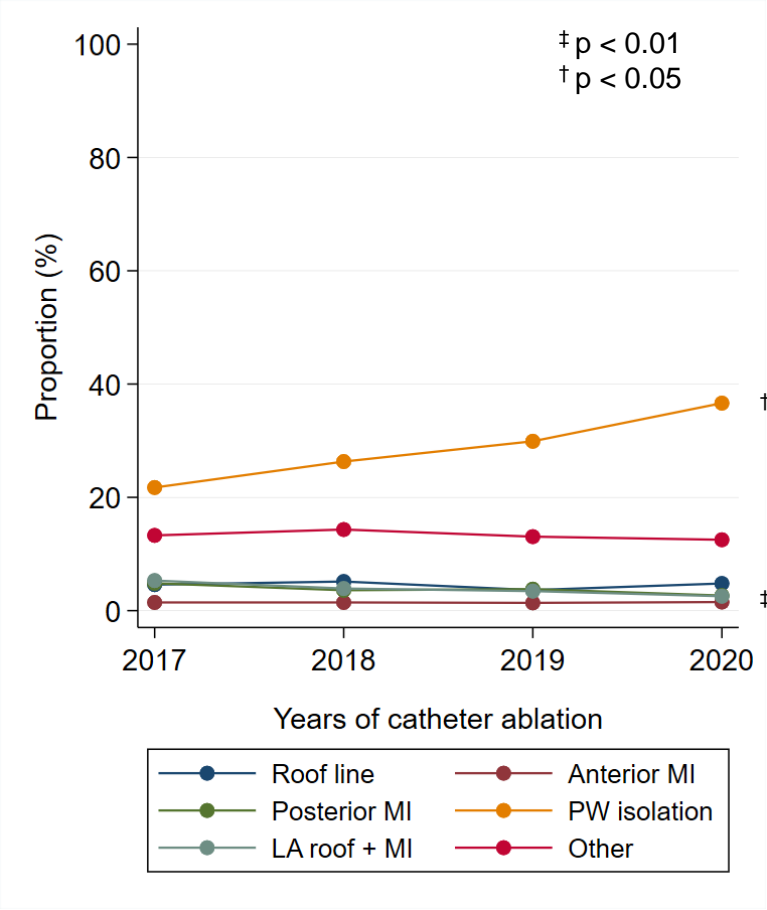

Supplement: Supplementary file 8 — Figure S8: Annual tendency of LA linear ablation in third or later ablation procedure. (A) LA posterior wall isolation showed an annual tendency to increase. In contrast, roof + MI showed an annual tendency to decrease over time. (B) In pAF, there was no annual tendency to increase or decrease for any strategy. (C) In perAF, LA posterior wall isolation showed an annual tendency to increase. In contrast, roof + MI showed an annual tendency to decrease over time. LA: left atrial, MI: mitral isthmus, pAF: paroxysmal atrial fibrillation, perAF: persistent atrial fibrillation, PW: posterior wall. [file JOA3-41-e70200-s005.pdf]
