# Supplementary material for: Real‐World Outcomes of Repeat Ablation Strategies for Atrial Fibrillation: Insights From the Japanese Catheter Ablation Registry
Source: J Arrhythm. 2025 Sep 22;41(5):e70200. doi: 10.1002/joa3.70200 (PMC12454675; doi:10.1002/joa3.70200)
Supplement: Supplementary file 5 — Figure S5: Freedom from AF recurrence stratified by the details of additional ablation. (A) In patients with pAF and other than additional ablation only, there was no significant difference in AF recurrence rate among groups. (B) In patients with pAF and additional ablation only, there was no significant difference in AF recurrence rate between patients with LA linear ablation and patients with other LA ablation. (C) In patients with perAF and other than additional ablation only, there was no significant difference in AF recurrence rate among groups. (D) In patients with perAF and additional ablation only, there was no significant difference in AF recurrence rate between patients with LA linear ablation and patients with other LA ablation. AF: atrial fibrillation, pAF: paroxysmal atrial fibrillation, perAF: persistent atrial fibrillation, PVI: pulmonary vein isolation, LA: left atrial. [file JOA3-41-e70200-s003.pdf]

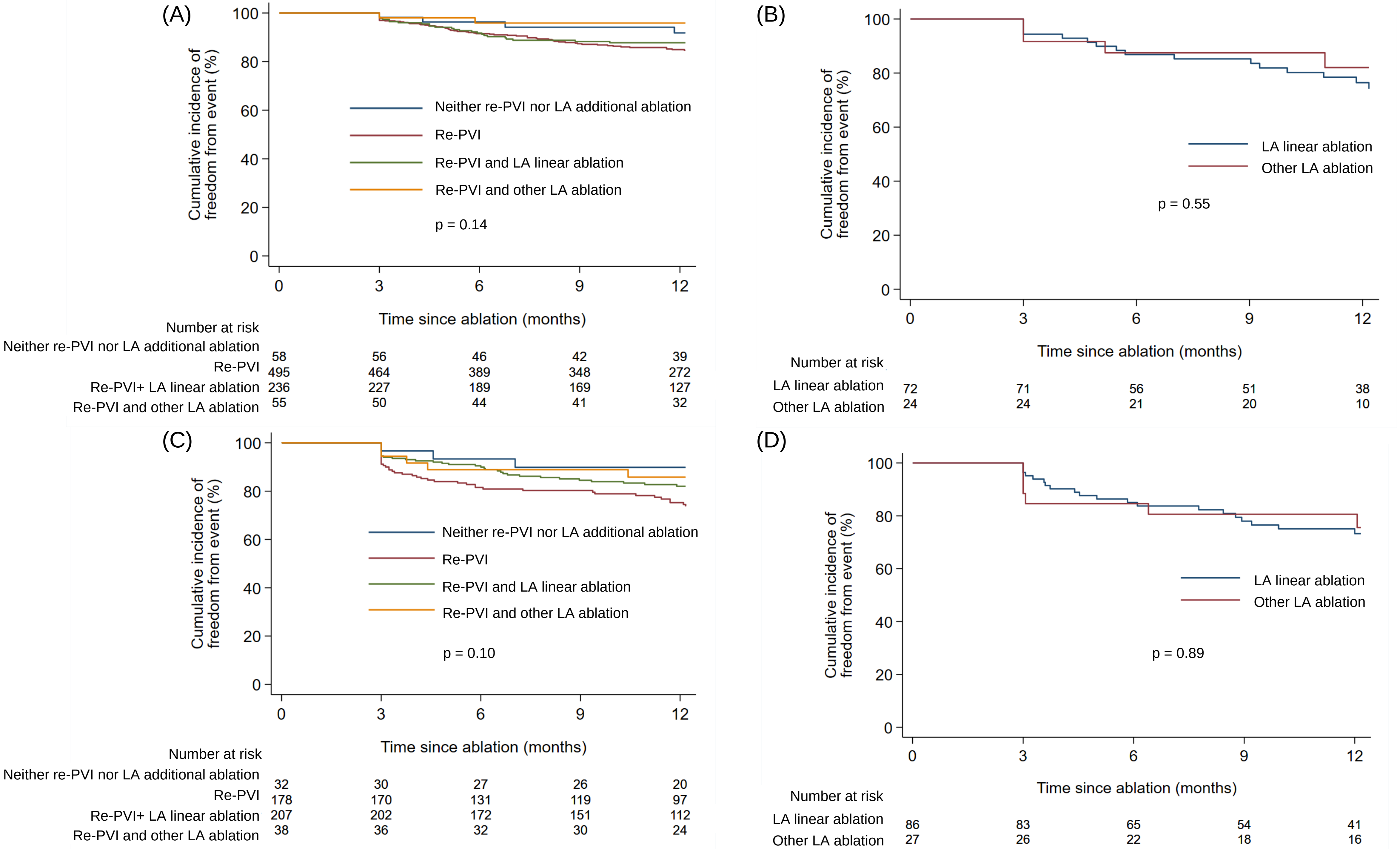

(A)
Cumulative incidence of freedom from event (%)
100
80
60
40
20
0
Neither re-PVI nor LA additional ablation
Re-PVI
Re-PVI and LA linear ablation
Re-PVI and other LA ablation
p = 0.14
0
3
6
9
12
Time since ablation (months)
Number at risk
Neither re-PVI nor LA additional ablation 58 56 46 42 39
Re-PVI 495 464 389 348 272
Re-PVI+ LA linear ablation 236 227 189 169 127
Re-PVI and other LA ablation 55 50 44 41 32
(B)
Cumulative incidence of freedom from event (%)
LA linear ablation
Other LA ablation
p = 0.55
Time since ablation (months)
Number at risk
LA linear ablation 72 71 56 51 38
Other LA ablation 24 24 21 20 10
(C)
Cumulative incidence of freedom from event (%)
Neither re-PVI nor LA additional ablation
Re-PVI
Re-PVI and LA linear ablation
Re-PVI and other LA ablation
p = 0.10
Time since ablation (months)
Number at risk
Neither re-PVI nor LA additional ablation 32 30 27 26 20
Re-PVI 178 170 131 119 97
Re-PVI+ LA linear ablation 207 202 172 151 112
Re-PVI and other LA ablation 38 36 32 30 24
(D)
Cumulative incidence of freedom from event (%)
LA linear ablation
Other LA ablation
p = 0.89
Time since ablation (months)
Number at risk
LA linear ablation 86 83 65 54 41
Other LA ablation 27 26 22 18 16
