# Supplementary material for: Real‐World Outcomes of Repeat Ablation Strategies for Atrial Fibrillation: Insights From the Japanese Catheter Ablation Registry
Source: J Arrhythm. 2025 Sep 22;41(5):e70200. doi: 10.1002/joa3.70200 (PMC12454675; doi:10.1002/joa3.70200)
Supplement: Supplementary file 6 — Figure S6: Freedom from AF recurrence stratified by details of LA linear ablation. (A) In patients with pAF and other than LA linear ablation only, there was no significant difference in AF recurrence rate among groups. (B) In patients with pAF and LA linear ablation only, there was no significant difference in AF recurrence rate between patients with LA posterior wall isolation and patients with other LA linear ablation. (C) In patients with perAF and other than LA linear ablation only, there was no significant difference in AF recurrence rate among groups. (D) In patients with perAF and LA linear ablation only, there was no significant difference in AF recurrence rate between patients with LA posterior wall isolation and patients with other LA linear ablation. AF: atrial fibrillation, pAF: paroxysmal atrial fibrillation, perAF: persistent atrial fibrillation, LA: left atrial. [file JOA3-41-e70200-s008.pdf]

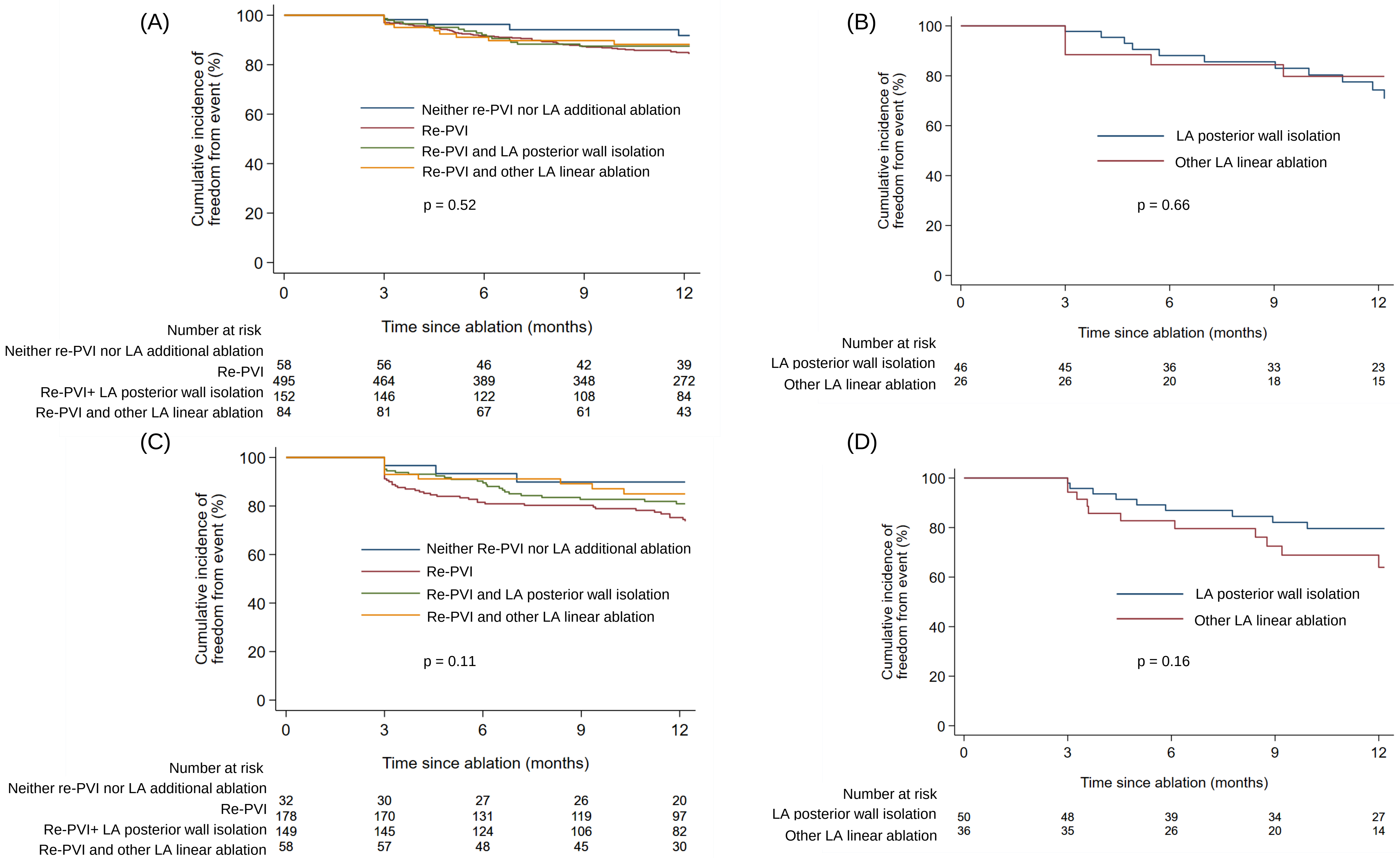
(A)
Cumulative incidence of freedom from event (%)
100
80
60
40
20
0
Neither re-PVI nor LA additional ablation
Re-PVI
Re-PVI and LA posterior wall isolation
Re-PVI and other LA linear ablation
p = 0.52
0 3 6 9 12
Time since ablation (months)
Number at risk
Neither re-PVI nor LA additional ablation 58 56 46 42 39
Re-PVI 495 464 389 348 272
Re-PVI+ LA posterior wall isolation 152 146 122 108 84
Re-PVI and other LA linear ablation 84 81 67 61 43
(B)
Cumulative incidence of freedom from event (%)
100
80
60
40
20
0
LA posterior wall isolation
Other LA linear ablation
p = 0.66
0 3 6 9 12
Time since ablation (months)
Number at risk
LA posterior wall isolation 46 45 36 33 23
Other LA linear ablation 26 26 20 18 15
(C)
Cumulative incidence of freedom from event (%)
100
80
60
40
20
0
Neither Re-PVI nor LA additional ablation
Re-PVI
Re-PVI and LA posterior wall isolation
Re-PVI and other LA linear ablation
p = 0.11
0 3 6 9 12
Time since ablation (months)
Number at risk
Neither re-PVI nor LA additional ablation 32 30 27 26 20
Re-PVI 178 170 131 119 97
Re-PVI+ LA posterior wall isolation 149 145 124 106 82
Re-PVI and other LA linear ablation 58 57 48 45 30
(D)
Cumulative incidence of freedom from event (%)
100
80
60
40
20
0
LA posterior wall isolation
Other LA linear ablation
p = 0.16
0 3 6 9 12
Time since ablation (months)
Number at risk
LA posterior wall isolation 50 48 39 34 27
Other LA linear ablation 36 35 26 20 14
